# Metabolomic Effects of Liraglutide Therapy on the Plasma Metabolomic Profile of Patients with Obesity

**DOI:** 10.3390/metabo14090500

**Published:** 2024-09-17

**Authors:** Assim A. Alfadda, Anas M. Abdel Rahman, Hicham Benabdelkamel, Reem AlMalki, Bashayr Alsuwayni, Abdulaziz Alhossan, Madhawi M. Aldhwayan, Ghalia N. Abdeen, Alexander Dimitri Miras, Afshan Masood

**Affiliations:** 1 Proteomics Resource Unit, Obesity Research Center, College of Medicine, King Saud University, Riyadh 11461, Saudi Arabia; helkamel@ksu.edu.sa (H.B.); afsmasood@ksu.edu.sa (A.M.); 2 Strategic Center for Diabetes Research, College of Medicine, King Saud University, Riyadh 11461, Saudi Arabia; 3 Department of Medicine, College of Medicine, King Saud University, Riyadh 11461, Saudi Arabia; 4 Metabolomics Section, Department of Clinical Genomics, Center for Genomics Medicine, King Faisal Specialist Hospital and Research Centre, Riyadh 11211, Saudi Arabia; aabdelrahman46@kfshrc.edu.sa (A.M.A.R.); rgalmalki@kfshrc.edu.sa (R.A.); 5 Corporate of Pharmacy Services, King Saud University Medical City, Riyadh 11461, Saudi Arabia; balsuwayni@ksu.edu.sa; 6 Department of Clinical Pharmacy, College of Pharmacy, King Saud University, Riyadh 11461, Saudi Arabia; alhossan@ksu.edu.sa; 7 Department of Community Health Science, Clinical Nutrition, College of Applied Medical Sciences, King Saud University, Riyadh 11461, Saudi Arabia; maldhwayan@ksu.edu.sa (M.M.A.); gabdeen@ksu.edu.sa (G.N.A.); 8 Section of Investigative Medicine, Division of Diabetes, Endocrinology and Metabolic Medicine, Hammersmith Hospital, Imperial College London, London SW7 2AZ, UK; a.miras@imperial.ac.uk; 9 School of Medicine, Ulster University, Derry BT1 6DN, UK

**Keywords:** obesity, liraglutide, metabolomics, mass spectrometry, arachidonic acid, biomarkers, oxidized lipids

## Abstract

Background: Liraglutide, a long-acting glucagon-like peptide-1 receptor agonist (GLP1RA), is a well-established anti-diabetic drug, has also been approved for the treatment of obesity at a dose of 3 mg. There are a limited number of studies in the literature that have looked at changes in metabolite levels before and after liraglutide treatment in patients with obesity. To this end, in the present study we aimed to explore the changes in the plasma metabolomic profile, using liquid chromatography-high resolution mass spectrometry (LC-HRMS) in patients with obesity. Methods: A single-center prospective study was undertaken to evaluate the effectiveness of 3 mg liraglutide therapy in twenty-three patients (M/F: 8/15) with obesity, mean BMI 40.81 ± 5.04 kg/m^2^, and mean age of 36 ± 10.9 years, in two groups: at baseline (pre-treatment) and after 12 weeks of treatment (post-treatment). An untargeted metabolomic profiling was conducted in plasma from the pre-treatment and post-treatment groups using LC-HRMS, along with bioinformatics analysis using ingenuity pathway analysis (IPA). Results: The metabolomics analysis revealed a significant (FDR *p*-value ≤ 0.05, FC 1.5) dysregulation of 161 endogenous metabolites (97 upregulated and 64 downregulated) with distinct separation between the two groups. Among the significantly dysregulated metabolites, the majority of them were identified as belonging to the class of oxidized lipids (oxylipins) that includes arachidonic acid and its derivatives, phosphorglycerophosphates, N-acylated amino acids, steroid hormones, and bile acids. The biomarker analysis conducted using MetaboAnalyst showed PGP (a21:0/PG/F1alpha), an oxidized lipid, as the first metabolite among the list of the top 15 biomarkers, followed by cysteine and estrone. The IPA analysis showed that the dysregulated metabolites impacted the pathway related to cell signaling, free radical scavenging, and molecular transport, and were focused around the dysregulation of NF-κB, ERK, MAPK, PKc, VEGF, insulin, and pro-inflammatory cytokine signaling pathways. Conclusions: The findings suggest that liraglutide treatment reduces inflammation and modulates lipid metabolism and oxidative stress. Our study contributes to a better understanding of the drug’s multifaceted impact on overall metabolism in patients with obesity.

## 1. Introduction

Tackling an ever-increasing rate of obesity and reducing its associated comorbidities is a major concern of the health community. Although lifestyle interventions (diet and exercise) have significant effects on weight management, achieving long-term success at weight loss is challenging, and the prevalence of obesity continues to rise worldwide. The 2023 World Obesity Atlas report states that 38% (one in four people) of the world’s population is presently classified as overweight or obese, with a body mass index (BMI) exceeding 25 kg/m^2^. It is projected that, by 2035, this prevalence will increase to 51% [1]. Not only is obesity a health challenge, but it is also a known risk factor strongly associated with the development of numerous other chronic health disorders, including diabetes, hypertension, dyslipidaemia, metabolic-associated fatty liver disease (MAFLD), sleep disorders, cardiovascular disease (CVD), and cancers [2]. The development of these comorbid conditions negatively affects an individual’s health, eventually leading to a decreased lifespan or accelerated ageing. Although obesity is simplistically described as an excessive accumulation of fat in the body, its pathophysiology is more complex, with underlying low-grade chronic inflammation, oxidative stress and insulin resistance as important components [3].

The medical management of obesity has taken precedence as a mode for weight management with the advent of glucagon-like peptide-1 (GLP-1) receptor agonists, which have proven to be effective in inducing weight loss and maintaining it. Liraglutide is a long-acting GLP-1 receptor agonist with 97% similarity to the native mature GLP-1 hormone. The added value of liraglutide as a pharmacological approach for obesity management has been established through several randomized clinical trials. The use of liraglutide at a dose of 3 mg is associated with clinically meaningful weight loss, where, on average, 63.2% of the patients studied were reported to lose ≥5% of their body weight in the SCALE obesity randomized control trial (RCT) [4,5,6,7]. Along with improvements in glycaemic control and weight loss, liraglutide is also known to increase insulin response and secretion, improve pancreatic beta-cell function and lipid metabolism, and reduce cardiovascular risk factors [8,9].

The recent decade has seen an increase in the use of different ‘omics platforms’ in clinical studies for risk stratification and the monitoring of treatment, to identify underlying disease pathophysiology and molecular alterations in proteins and metabolites. Clinical metabolomics has been employed for the discovery of novel disease biomarkers, the diagnosis of known diseases, and the understanding or rationalization of disease mechanisms [10,11]. Metabolomic profiling of patients with obesity associated with diabetes [12], insulin resistance [13,14], atherosclerosis [15], metabolic syndrome [16,17], and bariatric surgery [18] has been performed previously. A common factor among these conditions is the combined dysregulation of glucose, plasma free fatty acids, and increased intracellular lipids, which alters insulin sensitivity, contributes to increased insulin resistance [19], and promotes inflammation, vascular dysfunction, and atherogenesis [20]. Perturbations in aromatic and branched-chain amino acids (BCAAs) and metabolites involved in nucleotide metabolism—such as urate and pseudouridine [21,22,23], acylcarnitines, and fatty acids [24,25]—have been identified previously. Studies on the effect of subcutaneously administered liraglutide on the metabolomic profile of patients with obesity are limited, and these studies were mainly conducted in animal models. In one study, NMR spectroscopy of urine from a diet-induced (DIO) obesity mouse model showed reduced levels of the end products of nicotinamide adenine dinucleotide metabolism and the TCA cycle, as well as increased dimethylglycine levels [26]. The metabolomic profiles of different tissues (hypothalamus, plasma, liver, and skeletal muscle) in a DIO mouse model with and without liraglutide treatment using liquid chromatography (LC)–tandem mass spectrometry (MS/MS) and gas chromatography (GC–MS/MS) revealed alterations in fatty acid, amino acid, and carbohydrate metabolism [27]. Our group previously identified significant changes in the proteomic profile associated with liraglutide (1.8 mg dose) treatment in patients with type 2 diabetes, which are involved in regulating insulin metabolism, reducing systemic chronic inflammation, and decreasing oxidative stress [28].

In the present study, we aimed to perform untargeted metabolomic profiling of changes in the plasma metabolome of patients with obesity before and after treatment with liraglutide. Identifying these metabolites using an untargeted metabolomics approach allowed us to characterize the altered systemic endogenous metabolites and their associated metabolic pathways, which could account for its therapeutic effects and associated weight loss. Studying these changes could provide a window into understanding the molecular effect of the treatment, and its possible use in the personalization of anti-obesity therapy in humans requires further research.

## 2. Materials and Methods

### 2.1. Ethical Considerations and Informed Consent

The study protocol and procedures used in the study were approved by the Institutional Review Board, College of Medicine, King Saud University (no. E-21-5853) prior to undertaking the study. All the participants provided written informed consent. The study was performed in accordance with the ethical standards of the Declaration of Helsinki and the universal International Conference on Harmonization-Good Clinical Practice Guidelines.

### 2.2. Study Subjects and Sample Collection

We recruited twenty-three patients with obesity who were followed by their primary physician in the obesity clinic at King Saud University Medical City. All patients had Class 2 obesity (BMI > 35 mg/kg^2^), with no associated comorbidities and were drug-naïve. Patients were assessed for the presence of any contraindications for liraglutide therapy, and were started on treatment if they had none. The treatment was initiated by their physician as a scaled-up dose from 0.6 mg to 3.0 mg once daily via subcutaneous injection over a period of 5 weeks. They were followed up with prospectively until they completed 12 weeks on liraglutide 3 mg. Blood samples were collected from the patients after a 10 h fast before initiating therapy (pre-treatment) and after completing 12 weeks of treatment (post-treatment). The plasma was separated via centrifugation (15 min, 3000× *g*), divided into several aliquots, and stored at −80 °C for further analysis. Laboratory investigations for fasting blood glucose, lipids, liver function, and CRP were carried out at both time points in the KKUH central laboratory as part of their clinical evaluation and management.

### 2.3. Biochemical Analyses

Biochemical analyses were carried out using a Dimension Xpand Plus integrated clinical chemistry autoanalyzer (Siemens Healthcare Diagnostics, Deerfield, IL, USA). HbA1c was analysed using high-performance liquid chromatography and ion-exchange chromatography (normal range 4.3–5.8%; Tosoh Bioscience, San Francisco, CA, USA).

### 2.4. Data Analyses

The laboratory data are presented as the means ± SDs. Statistical significance of the difference between two groups was analysed by a paired Student’s *t* test, with a value of *p*  <  0.05 considered statistically significant.

### 2.5. Sample Preparation for Metabolomics

Metabolite extraction was performed as described previously [29]. Briefly, a 100 μL aliquot of plasma was mixed with 900 μL of the extraction solvent, a 1:1 mixture of acetonitrile (ACN) and methanol (MeOH). Concurrently, quality control (QC) samples were generated by taking aliquots from all samples to verify system stability. The mixtures were agitated in a thermomixer (Eppendorf, Hamburg, Germany) at 600 rpm and kept at room temperature (RT) for one hour. Subsequently, the samples were centrifuged at 16,000 rpm and 4 °C for 10 min. After centrifugation, 950 μL of the resultant supernatant was transferred to a 1.5-mL Eppendorf tube and then subjected to complete evaporation using a SpeedVac system (Eppendorf, Hamburg, Germany). The dried samples were reconstituted with 100 μL of 50% mobile phases A and B (A: 0.1% formic acid in dH_2_O, B: 0.1% formic acid in 50% ACN:MeOH). This reconstitution was followed by brief vortexing and then introduction into the LC–MS system for analysis.

### 2.6. LC–MS Metabolomics

A Waters Acquity UPLC system coupled with a Xevo G2-S QTOF mass spectrometer and an electrospray ionization (ESI) source was used to explore the metabolic profile. The extracted metabolites were separated using an ACQUITY UPLC instrument with an XSelect column (100 × 2.1 mm 2.5 μm) (Waters Ltd., Elstree, UK). The mobile phase solvent A was 0.1% formic acid in dH_2_O, while solvent B consisted of 0.1% formic acid in 50% ACN:MeOH. The following gradient elution program was used: 0–16 min with 95-5% A, 16–19 min with 5% A, 19–20 min with 5–95% A, and 20–22 min with 5–95% A, all at a flow rate of 300 µL/min. MS spectra were obtained in both positive (ESI+) and negative (ESI−) electrospray ionization modes. The MS parameters were as follows: source temperature at 150 °C, desolvation temperature at 500 °C for both modes (ESI+, ESI−), capillary voltage at 3.20 kV (ESI+) or 3 kV (ESI−), cone voltage at 40 V, desolvation gas flow at 800.0 L/h, and cone gas flow at 50 L/h. The collision energies for low and high functions were set at off and at 10 V to 50 V, respectively, in MSE mode. The mass spectrometer was calibrated using sodium formate in the 100–1200 Da range. The data were collected using a Masslynx™ V4.1 workstation in continuum mode (Waters Inc., Milford, MA, USA).

### 2.7. Data and Statistical Analysis

The raw MS data were subjected to standard processing, starting with alignment based on *m*/*z* values, ion signal retention times, peak picking, and signal filtering based on peak quality using Progenesis QI v.3.0 software from Waters Technologies (Milford, MA, USA). Features identified in at least 50% of the samples were retained for subsequent analyses.

MetaboAnalyst version 5.0 (McGill University, Montreal, QC, Canada; (http://www.metaboanalyst.ca (accessed on 12 April 2024)) was used for the multivariate statistical analysis [30]. To select an appropriate statistical model, the datasets—comprising the compounds and their abundances—underwent mean normalization, Pareto scaling, and log transformation to ensure a normal distribution. These normalized datasets were then utilized to construct partial least squares-discriminant analysis (PLS-DA) and orthogonal partial least squares-discriminant analysis (OPLS-DA) models. The performance of the OPLS-DA models was assessed through the fitness of model (R2Y) and predictive ability (Q2) values using permutation validation with 100 samples [31].

Univariate analysis was performed with Mass Profiler Professional software v.15.0 (Agilent, Santa Clara, CA, USA). Significantly changed mass features were identified through volcano plot representation, employing a fold change (FC) thresh, (old of 1.5 and a *p* value < 0.05. Venn diagrams were created using MPP Software v. 15.0 from Agilent, Inc., Santa Clara, CA, USA. For the assessment of altered features, heatmap analysis was conducted using the Pearson distance measure as part of the Pearson similarity test.

### 2.8. Metabolite Identification

Progensis QI software v.3.0 was utilized to select and tag the significant features within each dataset for peak annotation. Metabolite chemical structures were determined by acquiring their accurate precursor masses, fragmentation patterns, and isotopic distribution using the Human Metabolome Database (HMDB) and METLIN MS/MS (https://metlin.scripps.edu (accessed on 1 May 2024)) [32]. Exogenous compounds such as drugs, food additives, and environmental compounds were excluded from the final list.

### 2.9. Bioinformatic and Network Pathway Analysis 

Bioinformatics analysis was conducted to identify pathways and biomarkers associated with post- and pre-liragutide treatment using MetaboAnalyst version 5.0. Furthermore, receiver operating characteristic (ROC) curves were generated using the PLS-DA approach for a comprehensive assessment to discover potential biomarkers. To explore the interactions among the identified metabolites and predict their involvement in biochemical pathways, the significantly differentially expressed metabolites identified in this study were analysed using Ingenuity Pathway Analysis (IPA) version Q2 2024 (https://digitalinsights.qiagen.com/IPA, accessed on 15 May 2024) (Qiagen, Aarhus, Denmark). Ingenuity pathway analysis was used to generate network maps for the interactions between the identified metabolites and to identify the associated molecular pathways influenced by these metabolites.

## 3. Results

### 3.1. Clinical and Biochemical Characteristics of the Study Group

The clinical and biochemical characteristics of the study population are listed in Table 1. The mean age of the participants was 36.0 ± 11.4 years. Significant changes (*p* < 0.05) in weight, BMI and HbA1c levels were noted.

The values are expressed as the means ± standard deviations (paired *t* tests, *p* values < 0.05). Abbreviations are as follows: BMI: body mass index, Hb: haemoglobin, WBC: white blood cell, RBC: red blood cell, PLT: platelet, ESR: erythrocyte sedimentation rate, ALT: alanine aminotransferase, AST: aspartate transaminase, HDL: high-density lipoprotein, LDL: low-density lipoprotein, HbA1C: glycated haemoglobin, CRP: C-reactive protein.

### 3.2. Mass Ion Detection and Metabolite Identification

A total of 20,168 mass ion features (10,840 positive and 9328 negative) were detected in ionization mode. After several filtration processes, including alignment, peak picking, and missing value removal, a filter with a cut-off percentage >80% was applied to all samples. The remaining 15,975 features were subjected to statistical analysis (*p* value ≤ 0.05 and FC 1.5), which revealed that 636 metabolites were significantly dysregulated, 233 of which were upregulated and 403 of which were downregulated (Supplementary Table S1). To confirm that all depicted data had a Gaussian distribution, the median, log-transformed, and normalized data were Pareto scaled to eliminate systemic variances. Among the 636 metabolites, only 398 were annotated using The Human Metabolome Database (HMDB), KEGG, MassBank, LipidMAPS, and METLIN MS/MS databases (Supplementary Table S2). Exogenous metabolites (i.e., drugs, drug metabolites, environmental exposures, etc.) were excluded, and 161 endogenous metabolites were identified in both groups (Supplementary Table S3).

### 3.3. Overview of the Post- and Pre-Liraglutide Treatment Groups

An overview of the significantly altered ions between the groups was generated using the PLS-DA model, which was generated to examine any sample clustering and group separation in the datasets and to identify any possible outliers, as shown in Supplementary Figure S1A. The OPLS-DA between the two groups indicated a significant metabolic difference between the post- and pre-liraglutide treatment groups, according to the fitness of the model (R2Y = 0.956) and its predictive ability (Q2 = 0.786) (Supplementary Figure S1B).

The volcano plot (*t* test (*p* value < 0.05) and fold change (FC cut-off of 1.5)) between the post- and pre-liraglutide-treated groups is shown in Supplementary Figure S2. The results revealed that, of the 636 dysregulated metabolites identified, 233 (red) were upregulated and 403 (blue) were downregulated in the post-liraglutide-treated group.

The metabolomics analysis revealed a significant (FDR *p*-value ≤ 0.05, FC 1.5) dysregulation of 161 endogenous metabolites (97 upregulated and 64 downregulated) with distinct separation between the post- and pre-treatment groups. Compared with the pre-treatment group, the metabolic plasma profile of the post-treatment group showed significant upregulation of cystine (FC 2.756; *p* ≤ 0.0001), estrone (FC 2.48; *p* = 0.004), 13E-tetranor-16-carboxy-LTE4 (FC 2.10; *p* = 0.023), diacylglycerol (DG) (18:3(10,12,15)-OH(9)/0:0/8:0) (FC 3.08; *p* = 0.002), angiotensinogen (FC 1.64; *p* = 0.04), 2-(4-methyl-1-piperazinyl), ethanamine (FC 3.026; *p* = 0.007), pyrogallol-2-O-glucuronide (FC 2.22; *p* = 0.003), CDP-DG(a-17:0/20:4–2OH) (FC 2.05; *p* = 0.026), alanylvaline (FC 1.72; *p* = 0.022), and Gly-Pro-Arg-Pro-Lys (FC 2.09; *p* ≤ 0.0001). In contrast, metabolites such as PGP(LTE4/i-20:0) (FC 2.52; *p* = 0.021), CE(LTE4) (FC 2.84; *p* = 0.019), N-palmitoyl phenylalanine (FC 1.86; *p* = 0.04), lysoPC (10:0/0:0) (FC 1.73; *p* = 0.001), and 2,3,4,5,6,7-hexahydroxyheptanoic acid (FC 2.99; *p* ≤ 0.0001) were significantly downregulated in post-liraglutide treatment groups (Supplementary Data S1).

### 3.4. Evaluation of Metabolite Biomarkers between the Pre- and Post-Liraglutide Treatment Groups

**Figure 1:**
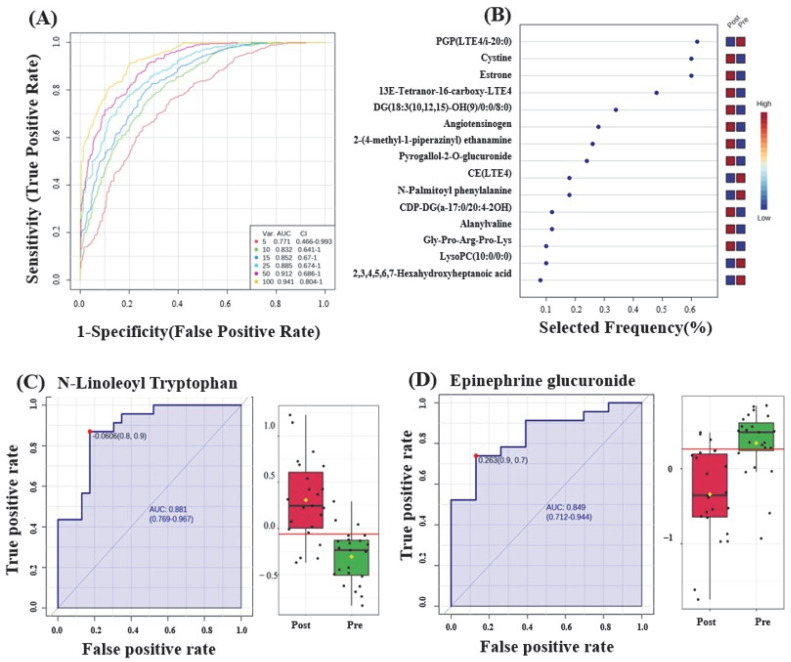
(**A**) Receiver operating characteristic (ROC) curve utilizing PLS-DA as the classification and feature ranking method. The top 15 variants had an area under the curve (AUC) of 0.852. (**B**) Frequency plot showing the top 15 significantly dysregulated metabolites in the pre- and post-liraglutide treatment groups. ROC curves are shown of individual metabolite biomarkers: (**C**) N-linoleoyl tryptophan, with an AUC of 0.881, and box plot (*p* ≤ 0.05 and fold change ≥ 1.5), where red represents the post-liraglutide treatment group and green represents the pre-liraglutide treatment group; and (**D**) epinephrine glucuronide, with an AUC of 0.849, and box plot (*p* ≤ 0.05 and fold change ≥ 1.5), where red represents the post-liraglutide treatment group and green represents the pre-liraglutide treatment group.

The AUC of the exploratory ROC curve for the top 15 metabolites was 0.852, as shown in Figure 1A. OPLS-DA was used as a classification and feature ranking approach for the multivariate exploratory ROC analysis based on the common and significantly dysregulated metabolites identified between the pre- and post-liraglutide treatment groups (Figure 1B). From the top 15 metabolites, two metabolites—N-linoleoyl tryptophan (AUC = 0.881), whose expression was upregulated, and epinephrine glucuronide (AUC = 0.849), whose expression was downregulated—are shown in Figure 1C,D, respectively. Box and whisker plots for N-linoleoyl tryptophan are shown in Figure 1C. The green box indicates the pre-treatment groups, and the red box indicates the post-treatment group, with an FDR *p* ≤ 0.05 and a fold change ≥ 1.5. Box and whisker plots for epinephrine glucuronide are shown in Figure 1D, with a fold change ≥ 1.5 and FDR *p* ≤ 0.05; the green box denotes the pre-treatment group, and the red box denotes the post-treatment group.

### 3.5. Interaction Network Pathway Analysis of Differentially Regulated Metabolites

**Figure 2:**
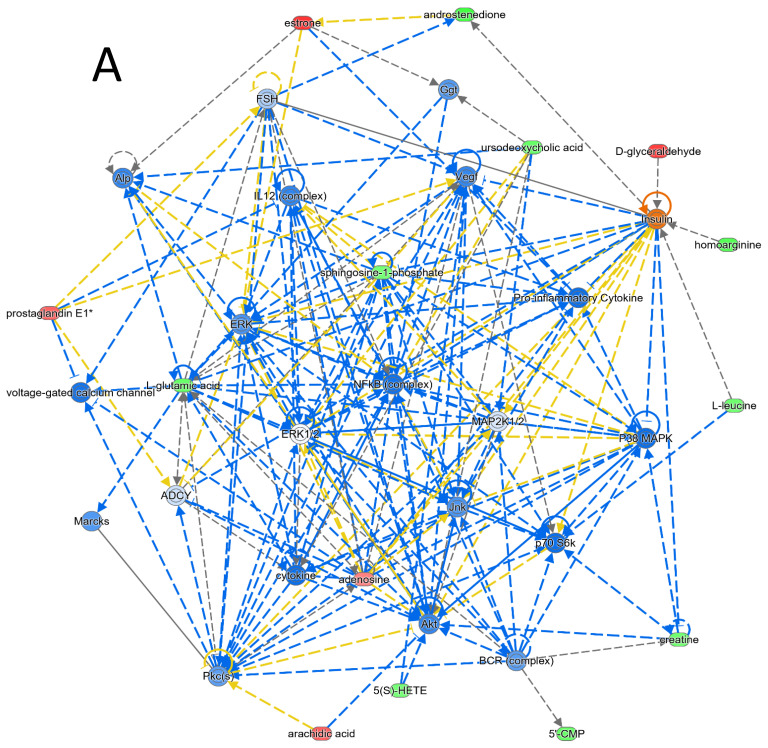

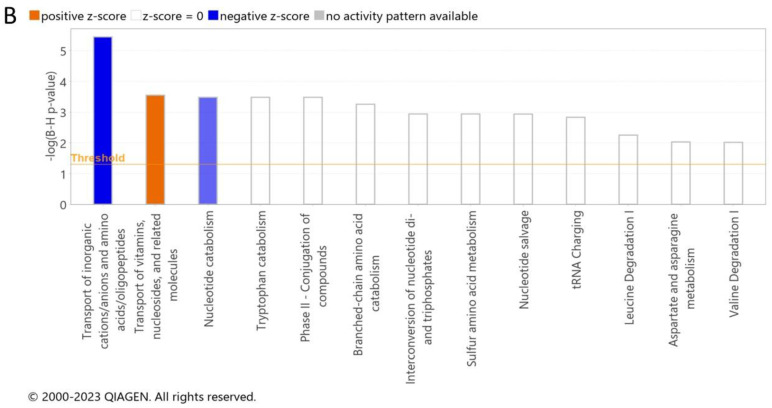
Schematic representation of the (**A**) highest scoring network pathways depicting the involvement of the differentially regulated metabolites between the pre- and post-liraglutide treatment groups. The dotted lines indicate indirect relationships, and the straight lines indicate direct relationships. The network pathways identified between the two groups were related to cell signalling, free radical scavenging, and molecular transport, with a score of 36 and 14 focus molecules (represented in bold Supplementary Table S4). The interaction networks were generated through IPA (QIAGEN, Aarhus, Denmark). (**B**) The top canonical pathways dysregulated after 12 weeks of treatment with liraglutide.

Bioinformatic analysis of the significantly differentially expressed metabolites was conducted using metaboanalyst (Supplementary Figure S3) and Ingenuity Pathway Analysis (IPA) version Q2 2024 to investigate the potential pathways related to pre- and post-treatment with liraglutide in patients with obesity. The pathway identified using Metaboscape with the greatest impact was related to arachidonic acid metabolism. The highest-scoring interaction network of the significant metabolites identified between the two groups was related to immunological diseases, inflammatory diseases, and the inflammatory response (Figure 2A). The network pathway IPA revealed that five metabolites in our dataset regulated EGFR and that four regulated insulin. Moreover, the five most significantly enriched canonical pathways between the pre- and post-treatment groups included the following: transport of inorganic cations/anions and amino acids/oligopeptides: 1.75 × 10 ^−8^ (with an overlap of 3.4% 6/177); transport of vitamins, nucleosides, and related molecules: 2.75 × 10 ^−6^ (with an overlap of 4.0% 4/100); nucleotide catabolism: 6.07 × 10 ^−6^ (with an overlap of 3.3% 4/122); and tryptophan catabolism: 7.05 × 10 ^−6^ (with an overlap of 7.9% 3/38) (Figure 2B).

## 4. Discussion

In the present study we carried out an untargeted metabolomic analysis of plasma samples from patients with obesity to investigate changes in the plasma metabolome before and after 12 weeks of treatment with 3 mg of the GLP-1 RA liraglutide. Clinically, we noted significant and clinically meaningful changes in the weight, BMI, and HbA1c levels of the patients at 12 weeks post treatment. Our findings are in line with RCT data from the SCALE Obesity and Prediabetes trial [7]. The levels of other biochemical parameters, including cholesterol, LDL, triglycerides, insulin, and CRP were lowered post treatment, although these did not reach statistical significance. The metabolomic profiling of patients with obesity between the pre- and post-treatment groups revealed statistically significant changes in 161 endogenous metabolites (97 and 64 were upregulated and downregulated, respectively). Bioinformatic network pathway analysis of the dysregulated metabolites revealed a significant impact on the arachidonic acid and riboflavin metabolic pathways, which are potential underlying metabolic pathways altered with weight loss and treatment. The altered metabolites showed high interconnectivity, and were related to cell signalling, molecular transport, and vitamin and mineral metabolism. For the first time, we report modulations in the levels of different lipid species, acylcarnitines, amino acids, and nucleotides in a longitudinal cohort of obese patients following a 12-week treatment with liraglutide 3 mg. These findings may suggest targets for GLP-1 RA therapy that could serve as biomarkers for assessing the effectiveness of weight loss and treatment response beyond glycaemic and weight control.

### 4.1. Dysregulated Lipid Species Demonstrated a Distinct Pattern Associated with Treatment

A large number of lipid species were dysregulated between the pre- and post-treatment groups. These included diglycerides, oxidized lipids (arachidonic acid (AA) and its metabolites, glycerophospholipids (GP)), ceramides, sterols, and bile acids. Lipids are important constituents of the cell membrane [33] and mediators of cell signalling and inflammation, with proven roles in health and disease states [34,35,36]. The dysregulation of oxylipins (oxidized lipids)—including PL and glycerol PL, steroid hormones and bile acids—was observed in our present study.

### 4.2. Modulation of Metabolic Pathways Related to Oxidized Lipids, Arachidonic Acid, Leukotrienes (LTs), and Prostaglandins (PGs)

Our metabolomic analysis revealed dysregulation of the metabolism of arachidonic acid (AA), an omega 6 long chain (C20:4) polyunsaturated fatty acid, and its related metabolites. AA metabolism yields a wide spectrum of biologically active metabolites, such as prostaglandins (PGs), thromboxanes (TXs), lipoxins (LXs), and leukotrienes (LTs), depending on the involvement of the cyclooxygenase (COX), lipoxygenase (LOX), or cytochrome P450 (CYP450) enzyme systems [37]. AA and eicosanoids regulate diverse physiological and pathological processes, including inflammation, oxidative stress, insulin secretion, the progression of obesity, diabetes, hepatic fibrosis, and cancers. Adipose tissue is a known site for eicosanoid synthesis, and an increase in its mass, as observed in patients with obesity or inflammation, positively impacts the levels of these metabolites. Previous studies in humans and animal models have shown that the 5-LOX/LT pathway and, more recently, the 12/15-lipoxygenase pathway are linked to the pathogenesis of obesity and its associated metabolic disorders [38,39], as well as to insulin resistance and fatty liver disease [40,41,42]. Interestingly, AA, HETE, and its isoforms showed a linear association with BMI and WC, and are considered signature metabolites of obesity overlapping with IR [43,44]. Among the PGs, PGE1 has been shown to inhibit platelet aggregation, mediate inflammation [45], and decrease lipolysis [46], while PGF2b is an indicator of oxidative stress. We also identified another class of oxidized lipids or oxylipins—namely, glycerophospholipids (GPs)—which are recognized as signalling molecules linked to PGs in our study [21,40]. Several GPs that bind to PGs were significantly dysregulated between the two groups (PGP(a-25:0/LTE4), PE(PGF1alpha/P-18:0), PE(PGD1/20:0), PC(TXB2/18:1), and PC(15:0/PGJ2) were increased, while PGP(LTE4/i-20:0), PA(19:2/PGJ2), PC(P-18:1/PGE2), PE(24:1/LTE4), PGP(a-25:0/PGE1), PI(PGJ2/20:0), and PS(PGD1/20:4) were decreased). The synthesis of these GPs is also controlled enzymatically via the COX, LOX, and CYP450 systems, and by nonenzymatic oxidation due to increased oxidative stress [47]. Among these, PGP(LTE4/i-20:0), consisting of one chain of LTE4 and a chain of 18-methylnonadecanoyl, was identified as the top molecule in the biomarker analysis. A decrease in the level of PGP(LTE4/i-20:0) could be a potential marker for decreased oxidative stress after weight loss or post treatment. More mechanistic studies are required to understand the role of these oxidized lipids in weight loss.

### 4.3. Dysregulation of Steroid Hormones and Bile Acids Associated with Weight Loss and Liraglutide Treatment

A significant disturbance in metabolites involved in the sterol hormone and bile acid pathways was observed between the post-treatment and pre-treatment groups. Obesity, a well-established factor influencing steroid hormone levels, was found to impact several key hormones. Notably, the levels of androstenedione, androsterone, and methoxyestrone decreased, while the levels of estrone, a weak oestrogen, increased in the post-treatment group compared to those in the pre-treatment group. Androstenedione serves as a precursor for the synthesis of both androgens and oestrogens. The conversion to oestrogens, particularly estrone, is known to occur in adipose tissue through the enzymatic activity of aromatase [48,49,50]. This conversion was found to correlate with total body weight and BMI [51]. Oestrogens have been previously shown to enhance pancreatic β-cell function and improve insulin sensitivity in animal models. Additionally, studies indicate that oestrogens play a role in regulating energy metabolism and glucose homeostasis, preventing fat accumulation, and overcoming leptin resistance in female mice fed a high-fat diet [52]. Within our dataset, estrone emerged as one of the top 15 molecules in the biomarker analysis between the pre- and post-treatment groups.

Bile acid levels exhibited distinct changes between the pre- and post-treatment groups. Trihydroxy-5b-cholanoic acid increased, while tetrahydroxy-5β-cholan-24-oic acid, urobilinogen, ursodeoxycholic acid, and biotripyrrin-b decreased. Bile acids are increasingly recognized as pivotal regulators of systemic metabolism, acting not only in nutrient absorption but also as signalling molecules. Their interactions with receptors—such as the farnesoid X receptor (FXR) and the G-protein-coupled bile acid receptor 5—influence hormone secretion, cholesterol metabolism, and energy expenditure. Altered bile acid levels are linked to conditions like type 2 diabetes, and their increase has also been noted post-bariatric surgery, correlating with improvements in metabolic parameters. Modulating bile acid levels and signalling presents a promising therapeutic avenue for treating obesity, type 2 diabetes, and metabolic syndrome [53].

### 4.4. Dysregulated Amino Acids and N-Acyl Amides Demonstrate a Distinct Pattern Associated with Treatment

In our study, we found distinct changes in the regulation of amino acids, dipeptides, and tripeptides between the pre- and post-treatment groups. The levels of beta-citryl-l-glutamic acid, creatine, leucine, glutamic acid, glutaminyl-l-arginine, and threonyllysyl-l-prolyl-l-arginine were reduced, while those of cysteine, alanylvaline, and gly-pro-arg-pro-lys were elevated post-treatment. Notably, cysteine, alanylvaline, and gly-pro-arg-pro-lys were among the top 15 metabolites identified via biomarker analysis. Cystine is an oxidized dimeric form of the sulphur-containing amino acid cysteine, a precursor to glutathione, which is an important antioxidant that has been linked to fat mass and insulin resistance in obese patients [54]. ROC curve analysis revealed that cysteine was a sensitive marker, with an AUC of 0.83. The increase in cysteine levels may be related to the anti-inflammatory effects of liraglutide or to the increased fat mass of the cohort after treatment with liraglutide 3 mg. Additionally, we observed dysregulation of the levels of N-acyl amide (ten N-acetylated amino acids, including N-acetylaspartic acid and N-palmitoyl phenylalanine, decreased post-treatment, while the levels of six N-acyl amides, including tyrosine and tryptophan, increased) and endogenous fatty acid compounds synthesized in adipose tissue [55,56,57]. N-acyl amides play various roles in cardiovascular function, metabolic balance, and obesity. N-acetylaspartic acid, which participates in acetate storage and transportation, might affect lipid metabolism pathways by influencing acetyl-CoA synthesis. Dysregulation of these metabolites could notably affect lipid metabolism.

### 4.5. Bioinformatic Analysis of Metabolite Interactions

The significantly different metabolites identified in our study between the patients before and after treatment were related to interactions that influenced cell signaling, free radical scavenging, and molecular transport. A total of 14 metabolites with the highest connectivity identified in the dataset were focused on regulating the NF-κB pathway, mitogen-activated protein kinase (MAPK), PKc, the ERK pathway, insulin, proinflammatory cytokine production, and the VEGF pathway (Figure 2, Supplementary Table S4). The NF-κB and MAPK signalling cascades—which include extracellular signal-regulated kinase (ERK) 1/2, c-Jun N-terminal kinase (JNK), and p38 MAPK—are known for their involvement in mediating inflammation and insulin resistance [19,58,59]. The activity of the signalling pathways regulated by these metabolites decreased after liraglutide 3 mg treatment. Modulation or inhibition of these pathways could be a potential strategy for the treatment of inflammation [60]. The network pathway map points to an increase in anti-inflammatory effects associated with 3 mg liraglutide treatment.

## 5. Conclusions

Our current untargeted metabolomics-based approach revealed that liraglutide treatment alters the arachidonic acid pathway and fatty acid oxidation, modulating lipogenic pathways and reducing inflammation and oxidative stress. This study also revealed the potential benefits of gut hormone-based therapy and contributed to a holistic understanding of the multifaceted impact of this drug on underlying metabolic pathways.

## Figures and Tables

**Table 1:** The clinical and biochemical characteristics of the study participants before and after 12 weeks of treatment with the GLP-1 RA, liraglutide.

	Pretreatment (*n* = 23)	Posttreatment with 3 mg Liraglutide (*n* = 23)	*p* Value
Age (years)	36 ± 10.9 years		
Weight (kg)	114.62 ± 13.2	106.33 ± 11.32	<0.001
BMI kg/cm^2^	42.26 ± 4.8	39.36 ± 5.05	<0.001
ALT IU/L	31.47 ± 12.8	34.78 ± 12.1	0.569
AST IU/L	20.65 ± 8.2	17.33 ± 7.12	0.104
Albumin gm/L	37.45 ± 3.3	38.60 ± 2.2	0.716
Creatinine mmol/L	65.68 ± 15.6	70.09 ± 19.5	0.031
Glucose mmol/L	4.85 ± 0.62	4.84 ± 0.908	0.972
GGT unit/L	32.27 ± 22.1	29.54 ± 18.1	0.267
Corrected calcium	2.34 ± 0.07	2.35 ± 0.07	0.587
Hba1c in %	5.69 ± 0.4	5.33 ± 0.3	<0.001
Cholesterol mmol/L	4.89 ± 1.1	4.77 ± 1.0	0.447
HDL mmol/L	1.35 ± 0.3	1.31 ± 0.4	0.600
LDL mmol/L	2.84 ± 0.9	2.70 ± 0.9	0.330
Triglycerides mmol/L	1.23 ± 1.1	1.05 ± 0.6	0.279
Insulin mIU/L	20.02 ± 13.6	15.98 ± 5.6	0.209
CRP mg/L	8.78 ± 8.7	7.45 ± 6.6	0.071

## Data Availability

All the data generated or analysed in the current study are included in this article.

## Supplementary Materials

The following supporting information can be downloaded at: https://www.mdpi.com/article/10.3390/metabo14090500/s1, Supplementary Figure S1: (A) PLS-DA showing clear separation between the pre- and post-liraglutide treatment groups. (B) OPLS-DA showing clear separation between the two groups, indicating a significant metabolic difference between the pre- and post-liraglutide-treated groups. The robustness of the created models was evaluated by the fitness of the model (R2Y = 0.956) and predictive ability (Q2 = 0.786). Supplementary Figure S2. Volcano plot showing the significantly dysregulated metabolites between the pre- and post-liraglutide-treated groups (paired *t* test, *p* value ≤ 0.05, fold change (FC) 1.5). Among the 636 significantly dysregulated metabolites, 233 were upregulated (red), and 403 were downregulated (blue) after liraglutide treatment. Supplementary Figure S3. Pathway analysis of 162 significantly dysregulated endogenous metabolites showing altered arachidonic acid and riboflavin metabolism between the post- and pre-treatment groups using metaboanalyst (http://www.metaboanalyst.ca), accessed on 12 April 2024). Supplementary Table S1: Binary comparison of pre- and post-saxenda treated patients. (paired *t*-test, no correction *p*-value ≤ 0.05, Fold change 1.5). Supplementary Table S2: List of Exogenous and endogenous metabolites. Paired *t*-test, no correction *p*-value ≤ 0.05, FC 1.5. Supplementary Table S3: List of Endogenous metabolites. Paired *t*-test, no correction *p*-value ≤ 0.05, FC 1.5. Supplementary Table S4: List of the metabolites associated with top two network pathways using ingenuity pathway analysis.
