# Supplementary material for: Metabolomic Effects of Liraglutide Therapy on the Plasma Metabolomic Profile of Patients with Obesity
Source: Metabolites. 2024 Sep 17;14(9):500. doi: 10.3390/metabo14090500 (PMC11433991; doi:10.3390/metabo14090500)
Supplement: Supplementary file 1 [file metabolites-14-00500-s001.zip › metabolites-3194674-supplementary Table S1-S3.pdf]

Supplementary Table 1: Binary comparision of Pre- and Post-saxenda treated patients. (paired t.test, no correction p-value ≤ 0.05, Fold change 1.5)

Compound	Retention Time	Mass	p ([Post] Vs [Pre])	FC (abs) ([Post] Vs [Pre])	Log FC ([Post] Vs [Pre])	Regulation ([Post] Vs [Pre])
0.74_638.8333m/z	0.7395499	638.8333	0.04250225	1.6137241	0.6903939	up
0.76_447.9154n	0.75670004	446.90814	0.026359238	1.9439915	0.9590219	up
0.76_509.8870n	0.75670004	508.87967	0.016754359	1.8940355	0.9214633	up
0.77_328.8641m/z	0.77383375	328.8641	0.03521362	1.5004764	-0.5854206	down
0.77_396.8521m/z	0.77383375	396.8521	0.039948467	1.5007726	-0.5857054	down
0.79_1025.1398n	0.79098344	1046.1145	0.026427204	1.7485123	0.8061279	up
0.79_1025.9683m/z	0.79098326	1025.9683	0.027763532	1.8567151	0.8927524	up
0.79_1051.1194m/z	0.79098344	1051.1194	0.01564739	1.6154184	0.6919079	up
0.79_1054.9932m/z	0.79098326	1054.9933	0.013852546	1.7164438	0.7794226	up
0.79_1070.9734m/z	0.79098326	1070.9734	0.009127085	2.4714825	1.3053766	up
0.79_1077.0215m/z	0.79098326	1077.0215	0.027880667	1.9745481	0.9815225	up
0.79_448.8568m/z	0.79098326	448.85678	0.037312448	1.9533483	-0.9659492	down
0.79_500.8580m/z	0.79098326	500.85794	0.005885274	2.4133592	-1.2710426	down
0.79_516.8430m/z	0.79098326	516.843	0.03433037	1.7454882	-0.8036306	down
0.79_554.9806m/z	0.79098344	554.9806	0.011696158	2.772687	1.4712847	up
0.79_581.0289m/z	0.79098326	581.02893	0.02989658	2.0984395	1.0693169	up
0.79_615.0168m/z	0.79098326	615.01685	0.026623275	1.9627826	0.9729004	up
0.79_651.9800m/z	0.79098326	651.98	0.021888543	1.8175489	0.86199415	up
0.79_690.9585m/z	0.7909833	690.9585	0.049965903	1.7179227	0.7806651	up
0.79_704.5841m/z	0.79098344	704.5841	0.005909961	1.7639862	0.8188393	up
0.79_815.9948m/z	0.79098326	815.9948	0.037037127	1.9070449	0.9313388	up
0.79_843.9699m/z	0.7909833	843.9699	0.04249364	1.5928419	0.671603	up
0.79_850.9931m/z	0.79098326	850.9931	0.021394718	1.5549412	0.63686	up
0.79_866.9795m/z	0.79098344	866.9795	0.024525948	1.7323376	0.7927201	up
0.79_877.9494m/z	0.7909833	877.9494	0.044428974	1.6447862	0.7179001	up
0.79_880.0884m/z	0.79098344	880.0884	0.027328623	1.917659	0.9393462	up
0.79_880.9531m/z	0.79098344	880.9531	0.03604346	1.9398161	0.95591986	up
0.79_896.9109m/z	0.79098326	896.9109	0.009125661	2.151897	1.1056089	up
0.79_903.0203m/z	0.79098326	903.02026	0.009080958	1.7653048	0.8199173	up
0.80_353.1303m/z	0.80243313	353.13025	4.73E-04	1.5791446	-0.65914327	down
0.81_1119.0683m/z	0.8081168	1119.0684	0.04274396	1.7138749	0.77726185	up
0.81_1162.0375n	0.80811685	1161.0302	0.04440593	1.6082889	0.68552655	up
0.81_2136.2014n	0.80811685	1067.0934	0.030430008	2.0138452	1.0099528	up
0.81_308.0646n	0.80811685	329.0393	0.015644401	2.6603048	1.4115915	up
0.81_494.9403m/z	0.80811685	494.94034	0.018850114	1.7556744	0.81202525	up
0.81_639.9555m/z	0.80811685	639.95544	0.01205855	1.543447	0.626156	up
0.81_745.0380m/z	0.80811685	745.038	0.005795699	1.7017213	0.7669948	up
0.81_746.0401m/z	0.80811685	746.0401	0.028837526	1.8345251	0.8754066	up
0.81_842.9507m/z	0.80811685	842.95074	0.019070253	1.8036436	0.85091424	up
0.81_864.9394m/z	0.80811685	864.9394	0.02290878	2.1303558	1.0910944	up
0.81_885.9312m/z	0.80811685	885.9312	0.017055105	1.9892255	0.9922068	up
0.81_886.9318m/z	0.80811685	886.9318	0.031066554	1.5686615	0.649534	up
0.81_900.9123m/z	0.80811685	900.9123	0.043041978	1.7603896	0.8158947	up
0.81_962.8848m/z	0.80811685	962.8848	0.03705442	1.7557973	0.8121263	up
0.82_331.1472m/z	0.8195666	331.1472	0.006642255	1.6076539	-0.6849568	down
0.83_598.8536m/z	0.82526654	598.8536	0.001050863	1.9110119	-0.9343368	down
0.83_695.7793m/z	0.82526654	695.7793	0.004418047	1.625648	-0.70101494	down
0.83_706.7847m/z	0.82526654	706.7847	0.009891125	1.5121522	-0.59660333	down
0.83_729.7926n	0.82526654	710.7748	0.03613727	1.5452203	-0.6278125	down
0.83_738.7306m/z	0.82526654	738.7306	0.044565454	1.6342741	0.70865	up

0.83_798.6876m/z	0.82526654	798.6876	0.025152825	1.7790123	0.8310765	up
0.83_854.4030m/z	0.82526636	854.40295	0.023055285	1.7101332	0.7741087	up
0.84_250.8688m/z	0.8367161	250.86877	0.03756708	2.294894	1.1984274	up
0.84_636.5306m/z	0.84239966	636.53064	0.04326484	1.8706095	0.9035084	up
0.84_836.4166m/z	0.8423997	836.4166	0.01797596	2.0112078	1.0080621	up
0.84_955.4507m/z	0.8423997	955.45074	0.033752408	1.8635739	0.898072	up
0.86_1013.7042m/z	0.8595503	1013.7042	0.004201604	1.9800143	-0.9855108	down
0.86_326.7630m/z	0.8595503	326.76303	0.042646833	1.6931018	0.7596687	up
0.86_448.6756m/z	0.8595503	448.6756	0.01022058	1.7643027	0.8190981	up
0.86_484.8614m/z	0.8595503	484.86142	0.005604785	2.2282493	-1.1559106	down
0.86_640.7174m/z	0.8595503	640.7174	0.04868873	2.0327024	1.023399	up
0.86_826.3849m/z	0.8595503	826.3849	0.002166869	1.825159	0.8680221	up
0.88_1119.7274m/z	0.8766999	1119.7274	0.009940451	2.3734639	-1.246994	down
0.88_1166.6883m/z	0.87669986	1166.6884	0.019642236	2.406378	-1.2668632	down
0.88_830.0155m/z	0.87669986	830.01556	0.017400324	1.678762	0.74739766	up
0.88_833.1162m/z	0.8767001	833.11615	0.014524576	1.5202171	-0.6042773	down
0.88_846.1632m/z	0.8767001	846.1632	0.020493709	1.7327727	-0.7930824	down
0.88_908.7883m/z	0.87670004	908.7883	0.008940645	2.8806973	-1.526418	down
0.88_964.7675m/z	0.8767	964.7675	0.037907135	1.5166286	-0.60086787	down
0.88_992.1027m/z	0.87670004	992.10266	0.001814636	1.8614954	-0.896462	down
0.88_993.1227m/z	0.8767001	993.1227	0.02965676	1.6175915	-0.6938473	down
0.89_250.0969m/z	0.8938333	250.09692	0.015466255	1.7051629	-0.76990956	down
0.89_257.0583m/z	0.8938332	257.0583	0.014568478	1.6206636	-0.6965847	down
0.89_365.0767m/z	0.8938332	365.0767	0.011496306	1.7361885	-0.7959236	down
0.89_367.0738m/z	0.8938332	367.0738	0.007789504	2.280611	-1.1894203	down
0.89_388.9446m/z	0.8938332	388.94458	0.012042287	2.1803746	-1.124576	down
0.89_621.1872m/z	0.8938333	621.1872	0.029495804	1.8814951	-0.91187954	down
0.89_647.0065m/z	0.8938332	647.00653	0.03202862	1.6137573	0.69042355	up
0.91_160.0969m/z	0.9053664	160.0969	0.0245198	1.6515797	0.7238466	up
0.91_282.0698m/z	0.9109837	282.06985	0.009010732	1.8943775	-0.92172384	down
0.91_338.0742m/z	0.9109837	338.07416	0.004052527	1.6508809	-0.7232361	down
0.91_947.0894m/z	0.9109837	947.08936	0.0012865	1.6341016	-0.7084977	down
0.93_1011.1088m/z	0.9281162	1011.1088	0.001307741	1.8320079	-0.8734257	down
0.93_1028.3127m/z	0.9281162	1028.3127	0.013171285	2.47836	-1.3093857	down
0.93_1049.0968m/z	0.9281162	1049.0968	0.002128992	1.6504643	-0.7228719	down
0.93_1052.1274n	0.9281162	1051.1201	1.21E-04	1.7411362	-0.80002904	down
0.93_1110.2161m/z	0.9281162	1110.2162	0.005787833	1.7778118	-0.83010256	down
0.93_1132.1976m/z	0.9281162	1132.1976	0.014133515	1.6381215	-0.71204233	down
0.93_1150.1640m/z	0.9281162	1150.164	6.74E-04	1.7927905	-0.8422069	down
0.93_1153.1975m/z	0.9281162	1153.1975	4.04E-04	1.7347426	-0.79472166	down
0.93_1159.1631m/z	0.9281162	1159.1631	0.015532157	1.5259966	-0.6097517	down
0.93_1169.1682m/z	0.9281162	1169.1682	2.96E-04	1.7278743	-0.78899825	down
0.93_1180.1803m/z	0.9281162	1180.1803	0.028580176	1.5559403	-0.6377867	down
0.93_1188.1878m/z	0.9281162	1188.1877	9.22E-04	1.6181949	-0.6943854	down
0.93_1203.1831m/z	0.9281162	1203.1831	0.008765401	1.5885618	-0.6677212	down
0.93_243.0656m/z	0.9281162	243.06557	0.04233706	1.5268319	0.61054116	up
0.93_408.9362m/z	0.9281162	408.93616	0.014370052	2.583897	-1.3695486	down
0.93_714.0379m/z	0.9281162	714.0379	4.50E-04	1.9123107	-0.9353169	down
0.93_738.8253m/z	0.9281162	738.8253	0.011454045	1.7601916	-0.8157324	down
0.93_820.8054m/z	0.9281162	820.80536	0.008137291	2.0174174	-1.0125096	down
0.93_888.1593n	0.9281162	909.134	0.018276244	1.5631069	-0.64441645	down
0.93_893.0878m/z	0.9281162	893.08777	1.99E-04	1.6803863	-0.74879295	down
0.93_904.1351n	0.9281162	925.10974	0.005099631	1.597264	-0.6756028	down
0.93_931.1185m/z	0.9281162	931.1185	0.001464671	1.672791	-0.7422572	down

0.95_1021.7651m/z	0.94526666	1021.7651	0.01350621	2.2484143	-1.1689079	down
0.95_1143.7286m/z	0.94526666	1143.7286	0.00270535	1.8317269	-0.8732044	down
0.95_1173.7286m/z	0.94526666	1173.7286	0.005683812	1.6257043	-0.7010648	down
0.95_1199.7494m/z	0.9452666	1199.7494	0.037895855	2.3058412	-1.2052932	down
0.95_207.0535m/z	0.9452666	207.05353	0.001409817	1.722385	-0.7844077	down
0.95_267.0751m/z	0.94526666	267.07513	0.011192644	1.7230735	-0.78498423	down
0.95_305.1024m/z	0.94526666	305.10245	0.04416314	1.6279148	-0.70302516	down
0.95_636.0913m/z	0.94526666	636.09125	0.011745846	1.6093521	-0.68648	down
0.95_899.0725m/z	0.94526666	899.0725	2.10E-04	1.7086993	-0.77289855	down
0.95_950.1806m/z	0.94526666	950.1806	0.002306939	1.7947328	-0.8437691	down
0.95_973.1578m/z	0.94526666	973.1578	0.007821804	1.7981942	-0.8465488	down
0.96_1040.1155m/z	0.96239984	1040.1155	0.01636037	1.593341	-0.67205507	down
0.96_1079.1092n	0.96239984	1078.1019	0.003182135	1.6849067	-0.75266874	down
0.96_536.8926m/z	0.9623999	536.8926	0.01611156	1.6813837	-0.749649	down
0.96_794.0362m/z	0.96240014	794.03613	0.001642761	1.8846436	-0.9142917	down
0.98_1055.1106m/z	0.97954965	1055.1106	0.011283833	1.6841519	-0.75202227	down
0.98_1068.1013m/z	0.97954965	1068.1013	0.004046728	1.6940471	-0.76047397	down
0.98_1085.1050n	0.97954965	1084.0977	0.003681027	1.675113	-0.7442584	down
0.98_1089.0828m/z	0.97954965	1089.0828	2.38E-04	1.678769	-0.74740374	down
0.98_1115.0887m/z	0.97954965	1115.0887	5.34E-04	1.7315892	-0.7920967	down
0.98_188.0949m/z	0.97954965	188.09486	0.02957895	1.5344837	-0.61775327	down
0.98_486.9887m/z	0.9795497	486.98868	0.030196983	2.0333674	-1.023871	down
0.98_706.0562m/z	0.97954965	706.05615	0.003498317	1.7620008	-0.81721455	down
0.98_792.0484m/z	0.97954965	792.04834	0.012413733	1.5038278	-0.5886394	down
0.98_869.1170m/z	0.97954965	869.11694	0.004783341	1.8897228	-0.9181746	down
0.98_880.0925m/z	0.97954965	880.0925	0.008939275	1.6600211	-0.7312015	down
0.99_1108.2465m/z	0.9910836	1108.2465	0.038798824	1.5453365	0.627921	up
1.00_111.0066m/z	0.9966834	111.00657	0.03169592	2.3296943	1.2201406	up
1.00_175.0267m/z	0.9966837	175.02672	0.001579856	1.9681259	-0.97682256	down
1.00_446.1562m/z	0.9966835	446.15622	0.020330878	1.5241299	-0.60798585	down
1.00_835.1322m/z	0.99668366	835.1322	0.013266922	1.5905298	-0.6695074	down
1.00_906.2732m/z	0.99668336	906.2732	0.003422501	2.5100183	-1.3276979	down
1.01_612.6019m/z	1.0138333	612.6019	0.029629285	1.846377	-0.88469714	down
1.01_634.5352m/z	1.0138332	634.53516	0.009805991	1.8258076	-0.8685347	down
1.01_852.6563m/z	1.0138333	852.65625	0.03176036	1.880334	-0.9109889	down
1.04_578.5680m/z	1.0424168	578.568	0.010299797	2.638661	-1.3998059	down
1.11_489.1014m/z	1.1109837	489.1014	0.020047422	1.5976063	-0.6759119	down
1.11_512.0920n	1.1109837	511.0847	0.012363602	2.062181	-1.044171	down
1.13_378.9304m/z	1.1282998	378.93045	0.009939466	1.7447088	-0.8029862	down
1.23_622.9607m/z	1.2311505	622.9607	0.04752368	1.7044762	-0.7693285	down
1.32_243.0654m/z	1.3166838	243.06537	0.040647753	1.544766	-0.62738824	down
1.32_278.1273m/z	1.3166838	278.12726	0.003583125	1.9619426	-0.97228277	down
1.33_116.0705m/z	1.3338329	116.07052	0.013315325	1.8026835	-0.8501461	down
1.33_188.0946m/z	1.3338327	188.09459	0.004938704	1.8232882	-0.86654264	down
1.33_307.0377m/z	1.3338327	307.03775	0.028361771	1.6610477	-0.7320935	down
1.33_365.0372m/z	1.3338327	365.0372	0.009093703	1.8920474	-0.9199482	down
1.33_447.1633n	1.3338327	468.138	0.015473031	1.903036	-0.9283028	down
1.33_508.0585m/z	1.3338327	508.05853	0.011763437	1.9478915	-0.9619133	down
1.33_637.1682n	1.3338327	636.16095	0.007988762	2.3406994	-1.2269397	down
1.35_201.0393m/z	1.3509829	201.03926	0.011626238	1.5699596	-0.65072745	down
1.35_840.1422n	1.3509829	839.1349	0.024587281	1.6803811	-0.7487884	down
1.39_558.0098m/z	1.3852661	558.00977	0.018926786	1.6496145	-0.72212887	down
1.42_361.1960m/z	1.4197501	361.19598	0.04367991	1.7133973	-0.7768597	down
1.44_290.0912m/z	1.4366835	290.0912	5.06E-04	1.9973924	-0.9981178	down

1.51_636.1566m/z	1.5052494	636.1566	7.72E-04	3.065407	-1.6160786	down
1.57_128.0337m/z	1.573833	128.03369	0.02684131	1.7142011	-0.77753633	down
1.57_185.9950m/z	1.573833	185.99501	0.047704514	1.6237485	-0.69932824	down
1.57_240.0513m/z	1.573833	240.05135	0.01261215	2.1600568	-1.1110692	down
1.57_337.0290m/z	1.573833	337.02896	0.031213025	1.7404081	-0.7994256	down
1.57_339.0142m/z	1.573833	339.01416	0.001453779	1.8433726	-0.8823477	down
1.57_342.0435m/z	1.573833	342.04352	0.020188767	2.2722666	-1.1841321	down
1.59_346.0452m/z	1.5909661	346.0452	0.04056459	2.2478454	-1.1685429	down
1.64_196.0249m/z	1.6424001	196.02487	0.023533659	1.5408987	-0.62377197	down
1.73_342.0446m/z	1.7281007	342.04462	0.009920922	2.0599792	-1.0426297	down
1.75_164.9289m/z	1.7452497	164.9289	0.007862709	1.5450757	0.6276775	up
10.01_439.1648m/z	10.007912	439.16476	0.026315639	1.699408	-0.7650323	down
10.20_413.1671m/z	10.196481	413.16705	0.035825487	1.7455391	-0.8036726	down
10.59_400.1988m/z	10.590761	400.1988	0.0111428	1.5490886	0.63141966	up
10.66_452.1967m/z	10.659333	452.19666	0.00894521	1.5597111	-0.6412788	down
10.69_503.2624m/z	10.688255	503.26245	0.044839337	1.5775892	-0.6577215	down
10.78_210.0797m/z	10.779329	210.0797	0.049059168	1.5434341	-0.62614393	down
10.83_462.1792m/z	10.83075	462.17917	8.70E-04	1.6009436	-0.6789224	down
10.83_530.1678m/z	10.83075	530.1678	3.84E-05	1.833809	-0.87484336	down
11.10_414.3364m/z	11.099669	414.3364	0.014545579	2.0332472	-1.0237856	down
11.12_505.1507m/z	11.116463	505.15073	0.011022789	1.6299536	0.7048309	up
11.29_227.0406m/z	11.287902	227.04062	0.016012695	1.7454605	-0.8036077	down
11.41_366.1045m/z	11.407899	366.10452	0.010224287	2.0664306	-1.0471408	down
11.41_421.0628m/z	11.407899	421.06277	0.043698255	1.8805646	-0.91116583	down
11.46_255.1187m/z	11.459319	255.11873	3.81E-04	1.7439003	0.80231756	up
11.53_597.2396m/z	11.527886	597.2396	0.04434461	1.5994982	-0.67761934	down
11.63_405.1218m/z	11.630745	405.12183	0.034428537	2.749567	-1.4592044	down
11.73_337.1461m/z	11.733604	337.14612	0.0062594	1.7255516	-0.78705764	down
11.91_229.0553m/z	11.905023	229.05527	0.04773376	1.5931745	-0.67190427	down
12.09_748.2160m/z	12.087888	748.216	0.024048828	1.6268432	-0.7020752	down
12.43_209.0878m/z	12.430731	209.08778	0.022987157	1.7256304	0.7871235	up
12.43_399.1104m/z	12.430731	399.1104	0.030210761	1.5644299	-0.645637	down
12.47_357.1017m/z	12.465021	357.10175	0.014431571	1.5794089	-0.6593847	down
12.47_437.0560m/z	12.465021	437.05597	0.007247953	1.9360402	-0.95310885	down
12.47_506.0523n	12.465021	505.04504	0.038703278	1.8293104	-0.8712999	down
12.49_429.1149m/z	12.494053	429.1149	0.013680263	1.8826052	-0.91273046	down
12.62_461.2937m/z	12.619297	461.29373	1.42E-04	1.8414747	0.8808615	up
12.64_252.0762m/z	12.636448	252.0762	0.013621724	1.8224154	0.8658518	up
12.69_338.1516n	12.687866	337.14432	1.98E-04	1.5397713	-0.62271607	down
12.71_451.0743m/z	12.705017	451.07425	0.005072738	2.177781	-1.1228589	down
12.84_372.1379m/z	12.842156	372.13788	0.004224652	1.6618304	-0.7327732	down
13.04_358.1709m/z	13.0421505	358.17093	0.004394331	1.8362517	-0.8767638	down
13.06_613.3627m/z	13.059298	613.3627	0.006077101	2.492913	1.3178325	up
13.09_680.3454n	13.093587	701.32007	0.018106526	2.0513787	-1.0365938	down
13.37_304.0391m/z	13.367862	304.03912	0.029593918	1.7317793	-0.7922551	down
13.37_568.3171m/z	13.367862	568.31714	0.022122974	1.6413053	-0.71484363	down
13.39_429.2015m/z	13.385004	429.2015	0.005314179	1.5125154	-0.5969499	down
13.39_515.2888m/z	13.385004	515.2888	0.011773074	1.6023202	-0.6801625	down
13.45_507.2277m/z	13.453586	507.2277	0.03671311	1.973636	-0.98085594	down
13.48_394.2754m/z	13.482703	394.27536	0.046887442	1.6711013	0.7407992	up
13.54_528.1604m/z	13.539283	528.1604	0.012273391	2.3353496	-1.2236385	down
13.56_321.1404m/z	13.556434	321.14035	0.013511065	1.7795403	-0.8315046	down
13.59_459.1821m/z	13.590716	459.18213	0.029501164	1.8413815	-0.88078856	down
13.61_675.3271m/z	13.60785	675.32715	0.006813169	1.5390111	-0.6220037	down

13.63_457.1573m/z	13.625	457.15735	0.003915267	1.5377175	-0.6207904	down
13.64_443.1582m/z	13.64215	443.15817	0.002465464	1.6541858	-0.72612125	down
13.78_319.1352m/z	13.77929	319.13525	3.67E-05	1.563302	-0.6445965	down
13.79_309.1311m/z	13.785602	309.1311	0.015093282	1.5221747	0.60613394	up
13.80_318.2999m/z	13.802741	318.29993	5.11E-04	3.2768807	1.7123232	up
13.80_340.2821m/z	13.802742	340.28207	0.00761092	4.0843973	2.0301232	up
13.82_375.0744m/z	13.819884	375.07437	0.031694513	2.1199567	-1.0840348	down
13.83_497.2777m/z	13.830712	497.27774	0.004069313	1.532954	-0.61631435	down
13.85_204.5474m/z	13.854185	204.54736	0.049386613	1.5589714	0.6405945	up
13.85_384.3230m/z	13.854186	384.32303	0.006903545	1.7044718	0.7693247	up
13.94_313.1227m/z	13.939881	313.1227	0.014410984	1.5169365	0.6011607	up
13.95_329.2366m/z	13.950702	329.2366	0.033029057	1.6015576	0.67947567	up
14.05_655.2785m/z	14.047851	655.27844	0.00199152	1.5187913	-0.60292363	down
14.17_273.0691m/z	14.167848	273.06906	0.003168749	1.589615	0.6686774	up
14.17_406.1375m/z	14.167849	406.13754	0.040260307	2.4896703	-1.3159547	down
14.19_432.1503m/z	14.1849985	432.1503	0.006918493	1.522255	0.60621	up
14.26_239.0793m/z	14.259887	239.07932	0.010256497	2.0383556	1.0274057	up
14.29_226.9484m/z	14.294167	226.94843	0.018285286	1.7951835	0.84413135	up
14.31_638.1293n	14.311315	621.126	0.035161708	2.0749962	1.0531087	up
14.34_321.1498m/z	14.339289	321.14984	8.37E-05	1.5384978	-0.62152237	down
14.35_358.9795m/z	14.345702	358.97946	0.017810613	2.1454244	1.101263	up
14.37_460.2948m/z	14.373572	460.29483	0.004327671	1.8566513	-0.8927029	down
14.37_786.3977m/z	14.373573	786.39764	0.006659401	1.5210036	-0.60502356	down
14.39_429.2182m/z	14.390692	429.21817	5.50E-04	1.5283737	-0.61199737	down
14.39_499.2919m/z	14.390692	499.29184	0.001118688	1.5272464	-0.61093277	down
14.41_161.5019n	14.414266	365.03763	0.035561617	1.7552304	0.8116604	up
14.41_633.2566m/z	14.407843	633.2566	7.34E-04	1.61807	-0.694274	down
14.45_386.3094m/z	14.448549	386.30936	0.045934897	1.8626549	-0.8973604	down
14.49_408.1709m/z	14.493548	408.17093	8.41E-04	1.66203	-0.7329464	down
14.51_559.1171m/z	14.510699	559.11707	0.003329226	1.5500169	-0.6322839	down
14.53_290.1694n	14.534264	255.15555	0.046120957	1.5103858	0.59491706	up
14.53_435.2732m/z	14.52785	435.27316	5.66E-04	1.6587929	-0.7301337	down
14.56_253.1476m/z	14.562134	253.14755	5.67E-04	1.512098	-0.5965516	down
14.58_509.2363n	14.579266	508.22903	0.021347899	1.7573303	-0.81338537	down
14.60_163.5399m/z	14.602911	163.53987	0.015638122	1.8623451	0.8971204	up
14.65_375.1880m/z	14.647828	375.188	0.003978572	1.6744924	-0.7437238	down
14.66_321.1496m/z	14.664979	321.14957	5.72E-06	1.6040126	-0.68168545	down
14.76_346.3060m/z	14.757201	346.30603	0.010288355	1.6823361	0.7504659	up
14.77_220.6316m/z	14.774415	220.63156	0.01911984	1.5638534	0.64510524	up
14.77_366.2798m/z	14.774415	366.2798	0.02789115	1.5797948	0.6597371	up
14.78_385.2082m/z	14.784979	385.20825	0.005845116	1.5330954	-0.61644745	down
14.79_793.7934n	14.791558	280.2597	0.003437916	1.5832826	0.66291875	up
14.81_938.4975m/z	14.808696	938.49756	7.46E-09	8.125785	3.0225072	up
14.82_731.3701m/z	14.819269	731.37006	0.013894185	1.6032625	-0.68101066	down
14.83_268.2634m/z	14.8258295	268.26337	0.03036803	1.9327018	0.95061904	up
14.83_351.6282m/z	14.8258295	351.62817	0.025349632	1.7231792	0.78507274	up
14.84_295.1571m/z	14.83642	295.1571	9.76E-05	1.6547801	-0.72663957	down
14.91_170.0956m/z	14.911554	170.09557	0.04409952	1.5751343	0.65547484	up
14.91_205.1479m/z	14.911554	205.14789	0.04354215	2.1384678	1.0965774	up
14.91_286.3087m/z	14.911554	286.30875	0.024991505	2.8673744	1.5197303	up
14.96_521.2489m/z	14.956403	521.2489	0.04916055	1.5122046	-0.5966534	down
14.96_539.2901m/z	14.9564	539.2901	6.19E-04	1.9743012	-0.9813421	down
14.96_590.3112n	14.956403	589.3039	0.007035755	1.5572509	-0.63900137	down
14.96_616.2580n	14.956403	615.25073	4.18E-04	1.8491118	-0.8868324	down

14.97_283.1551m/z	14.973554	283.15515	6.47E-04	1.7100081	-0.7740032	down
14.97_324.1745n	14.973554	323.16727	4.89E-06	1.6877272	-0.7550817	down
15.00_445.2493m/z	15.002135	445.2493	0.004847816	1.6763755	-0.74534535	down
15.00_537.2599m/z	15.002134	537.25995	0.028688455	1.5049524	-0.58971786	down
15.01_483.3361m/z	15.008795	483.33615	0.014862713	1.7109269	0.7747781	up
15.02_558.2471n	15.019269	557.2398	0.00118408	1.72882	-0.78978765	down
15.02_574.2148n	15.019269	573.2076	0.003804523	1.8973418	-0.92397964	down
15.02_601.2080m/z	15.019268	601.208	0.00370947	1.9988497	-0.99917	down
15.02_612.2206m/z	15.019269	612.2206	0.001630891	1.7994195	-0.84753156	down
15.03_485.3411m/z	15.025939	485.3411	0.047622293	1.6458617	0.71884316	up
15.12_251.1447m/z	15.122126	251.14467	0.004216556	2.4895794	1.315902	up
15.14_333.2085m/z	15.1392765	333.20853	0.036308646	1.5187575	-0.6028915	down
15.16_751.3195m/z	15.163069	751.3195	0.01427484	1.8022197	0.8497749	up
15.16_769.4056m/z	15.156416	769.4056	1.79E-04	1.6166866	-0.69304	down
15.19_598.2491n	15.190695	597.2418	0.006471343	1.5369499	-0.6200701	down
15.28_773.3081m/z	15.276403	773.30817	0.02339146	1.7252537	0.7868085	up
15.29_311.2267m/z	15.293558	311.22668	0.013064166	1.6694373	-0.7393619	down
15.36_335.2245m/z	15.362121	335.2245	0.004685445	1.7073737	-0.7717789	down
15.39_474.2086m/z	15.385969	474.20862	0.026864978	1.522652	0.6065863	up
15.40_387.1330m/z	15.403119	387.13297	0.012375862	1.6478477	0.72058284	up
15.40_555.2002m/z	15.396399	555.20026	0.04734563	1.948762	-0.9625579	down
15.41_447.1353m/z	15.413553	447.1353	0.035010565	1.5735005	-0.65397763	down
15.44_574.1288m/z	15.437396	574.12885	0.019353442	1.8494316	0.887082	up
15.44_579.1145m/z	15.437398	579.11444	0.04055991	1.6754422	0.74454194	up
15.47_265.5763m/z	15.471681	265.5763	6.23E-04	1.5395107	0.6224719	up
15.49_563.1349m/z	15.4888315	563.1349	0.022859981	1.7143718	0.77768004	up
15.54_289.9208m/z	15.540267	289.92084	0.005882935	1.761599	-0.8168855	down
15.55_493.3555m/z	15.550681	493.3555	0.002789387	3.0884347	1.6268758	up
15.57_335.2258m/z	15.56783	335.22577	0.003637792	1.7899964	-0.83995664	down
15.57_651.2115m/z	15.574546	651.2115	0.03722198	3.1198432	-1.6414735	down
15.61_390.3349m/z	15.608827	390.33493	0.00757697	1.5923336	0.6711426	up
15.68_534.3608m/z	15.6774235	534.3608	0.02603176	1.771321	0.8248257	up
15.69_481.2820m/z	15.687826	481.282	0.007242425	1.6133037	-0.690018	down
15.69_549.2715m/z	15.687826	549.2715	0.003888055	1.5300717	-0.6135993	down
15.69_705.3742m/z	15.694574	705.37427	0.039644644	1.5216455	0.6056323	up
15.69_931.6036n	15.687831	930.5963	0.037818264	1.5493548	-0.63166755	down
15.77_407.2817m/z	15.773539	407.2817	0.024920136	1.6973178	0.7632567	up
15.77_454.2942n	15.773539	453.28693	0.03835908	1.5772458	0.6574075	up
15.77_535.2428m/z	15.773539	535.2428	4.64E-04	1.6597059	-0.7309276	down
15.77_555.2184m/z	15.773536	555.2184	0.025966574	2.3830142	1.2527876	up
15.77_579.2344m/z	15.773539	579.2344	0.001558833	1.6492906	-0.72184557	down
15.79_816.5678n	15.79171	839.557	0.019101772	2.2417703	1.1646384	up
15.84_391.2869m/z	15.842108	391.28693	0.009280845	1.7121753	-0.7758304	down
15.84_438.2993n	15.842108	437.29202	0.008242647	1.7699916	-0.8237425	down
15.84_505.2740m/z	15.842108	505.27402	0.026411226	2.1724513	-1.1193238	down
15.86_601.1945m/z	15.859251	601.19446	0.003799963	2.7321994	-1.4500628	down
15.88_533.2030m/z	15.876403	533.203	0.002804805	1.6622484	-0.73313594	down
15.91_376.2258m/z	15.910688	376.22577	0.001189998	1.5297576	-0.61330307	down
15.91_444.2175m/z	15.910688	444.21747	9.53E-04	1.5716854	-0.6523125	down
16.01_299.1872m/z	16.00783	299.1872	0.001942648	1.6002566	-0.67830324	down
16.02_265.1816m/z	16.024965	265.18158	0.024562092	1.5650793	-0.64623576	down
16.02_418.2231m/z	16.024965	418.2231	0.00759936	1.5094647	-0.59403706	down
16.02_785.4373m/z	16.024965	785.43726	0.003821015	1.5255303	-0.60931087	down
16.08_343.1593m/z	16.07641	343.15924	0.00757828	1.7102287	-0.77418923	down

16.12_615.4570m/z	16.117617	615.457	0.03837331	1.5917228	0.67058915	up
16.16_233.1561m/z	16.162111	233.15605	2.75E-04	1.52092	0.6049443	up
16.32_579.2293m/z	16.316404	579.22925	0.002177383	1.5804448	-0.66033065	down
16.33_1164.4924n	16.333534	1163.4852	0.043215875	1.6000043	-0.6780758	down
16.33_614.2424n	16.333538	613.2351	0.00924511	1.5265032	-0.6102306	down
16.33_644.2241n	16.333536	665.1987	0.003611861	2.0671008	-1.0476087	down
16.33_648.2781m/z	16.333536	648.27814	0.011459612	1.7223083	-0.78434336	down
16.33_671.2154m/z	16.333536	671.2154	0.031048154	1.7597153	-0.81534207	down
16.34_304.1091m/z	16.340572	304.1091	0.003897179	1.6457664	-0.71875954	down
16.37_640.4155m/z	16.367817	640.4155	0.026877115	1.6025022	-0.68032634	down
16.39_593.8947m/z	16.391973	593.89465	0.048561305	1.7778202	0.8301094	up
16.44_552.3567m/z	16.443419	552.3567	0.005036326	1.6464877	0.71939176	up
16.44_660.3228m/z	16.4364	660.3228	0.004585302	1.7606014	-0.8160683	down
16.45_799.4529m/z	16.453537	799.4529	5.95E-04	1.6428658	-0.7162146	down
16.46_388.5875m/z	16.460552	388.58746	0.011811323	1.9742776	0.98132485	up
16.46_788.4126n	16.460554	753.39874	0.01657602	1.796156	0.8449127	up
16.47_806.5851m/z	16.470686	806.58514	0.019195016	2.8465815	-1.5092304	down
16.49_525.3749m/z	16.487812	525.37494	0.001854017	1.8214052	-0.86505187	down
16.50_1082.7440m/z	16.504963	1082.744	0.03676738	2.276356	-1.1867262	down
16.54_738.5293m/z	16.539248	738.5293	0.004886203	1.5962318	-0.67467016	down
16.54_792.4900m/z	16.539248	792.49	0.007658529	1.9276978	-0.94687885	down
16.56_1047.5897m/z	16.556398	1047.5896	0.014281583	1.7224013	-0.78442127	down
16.56_519.2353m/z	16.556398	519.2353	0.044313807	1.8219109	-0.86545235	down
16.56_806.5120m/z	16.556398	806.51196	3.68E-04	1.7393585	-0.7985553	down
16.56_846.2257m/z	16.556398	846.2257	0.03682571	1.561219	-0.6426729	down
16.56_874.5028m/z	16.556398	874.5028	0.008360573	2.1651566	-1.1144713	down
16.56_934.5084m/z	16.556398	934.5084	0.008269195	1.9016781	-0.92727304	down
16.56_979.6014m/z	16.556398	979.60144	0.020224841	1.5144794	-0.59882194	down
16.61_820.5420m/z	16.607811	820.54205	0.021059707	1.6455169	-0.7185408	down
16.64_249.1539m/z	16.642094	249.15395	0.010280917	1.9302367	0.94877774	up
16.64_297.3345m/z	16.643538	297.33453	0.003630264	1.503608	-0.5884285	down
16.64_536.1330m/z	16.64354	536.13293	0.0495219	1.90667	-0.9310551	down
16.66_1072.4279n	16.660686	1055.4246	0.01840098	2.1112206	-1.0780773	down
16.66_378.2430m/z	16.659245	378.24304	0.001189006	1.549945	-0.632217	down
16.66_553.2026m/z	16.660686	553.2026	0.008314759	1.5490593	-0.63139236	down
16.66_593.1954m/z	16.660685	593.1954	0.042970274	1.7576227	-0.8136254	down
16.66_604.1865m/z	16.660683	604.1865	0.030956147	2.8268497	-1.4991951	down
16.70_673.5233m/z	16.695026	673.5233	0.034610577	1.6369916	0.71104693	up
16.70_693.2770m/z	16.695028	693.277	0.034590486	1.8112311	0.85697067	up
16.71_617.9044m/z	16.712177	617.9044	0.025193388	1.6993451	0.7649789	up
16.71_747.5481m/z	16.712177	747.54803	0.033190206	1.5898931	0.66892976	up
16.74_289.1851m/z	16.744974	289.1851	0.005953359	1.5225928	-0.60653013	down
16.80_310.3061m/z	16.797907	310.3061	0.03223362	1.9207435	0.9416648	up
16.80_386.1266m/z	16.796385	386.12662	2.00E-04	1.8166599	0.86128837	up
16.80_736.5182m/z	16.796385	736.5182	0.029406292	1.6123942	-0.6892045	down
16.80_872.2403m/z	16.796385	872.2403	0.014268116	1.539489	-0.6224516	down
16.82_728.4497m/z	16.815042	728.4497	0.04082652	1.5773349	0.657489	up
16.85_377.1641m/z	16.84782	377.16406	0.011275088	1.5844331	-0.6639667	down
16.85_846.5180m/z	16.849323	846.518	0.039184954	1.7001041	0.7656231	up
16.85_874.5622m/z	16.84782	874.5622	0.006024883	1.9570612	-0.96868885	down
16.87_838.6096m/z	16.86646	838.60956	0.017819095	1.6885568	0.7557907	up
16.88_1108.6643m/z	16.882095	1108.6643	0.002617257	1.5312817	0.6147397	up
16.88_543.2251m/z	16.883612	543.2251	0.00361613	2.1735206	1.1200337	up
16.88_800.6260m/z	16.883612	800.626	0.009610183	1.9609771	0.97157264	up

16.90_1190.6586m/z	16.899248	1190.6587	7.15E-04	1.6799607	0.7484275	up
16.90_564.5629m/z	16.899248	564.5629	0.017525991	1.5506222	0.63284725	up
16.95_986.5561m/z	16.950666	986.5561	0.03772573	1.7011077	-0.7664745	down
16.97_286.2368m/z	16.969406	286.23682	0.045250595	1.6048292	-0.6824197	down
16.97_320.2380n	16.96782	319.23077	0.00473435	1.5408114	-0.6236903	down
16.97_768.5570m/z	16.967817	768.55707	0.010740837	2.3707936	1.24537	up
16.99_720.9125m/z	16.98653	720.91254	0.037393313	1.5657783	0.6468799	up
17.02_685.9484m/z	17.020828	685.94836	0.025784882	1.5845753	0.6640962	up
17.02_714.8974m/z	17.020828	714.89734	0.014180744	1.5716515	0.6522813	up
17.05_239.1687m/z	17.04781	239.16869	4.41E-04	1.6811986	-0.74949014	down
17.06_205.2510m/z	17.06496	205.25096	0.047653724	1.5213182	0.60532194	up
17.06_758.5026m/z	17.06496	758.5026	1.11E-04	1.9888633	-0.99194413	down
17.07_413.2657m/z	17.07226	413.26575	0.02814506	1.5780616	0.6581535	up
17.09_113.1327m/z	17.089401	113.13271	0.044138268	1.7475712	0.80535126	up
17.09_464.7607m/z	17.089401	464.76065	3.64E-04	1.6098888	0.686961	up
17.10_984.6172m/z	17.099245	984.6172	0.04545859	1.6472609	-0.72006905	down
17.17_1030.5325m/z	17.167826	1030.5325	0.013111861	1.751642	0.80870795	up
17.17_535.2956m/z	17.167826	535.29565	3.75E-04	1.7262905	0.7876752	up
17.17_894.5496m/z	17.167826	894.5496	0.00778772	1.796045	0.8448234	up
17.18_831.0638m/z	17.17519	831.0638	0.031772695	1.6574695	0.7289823	up
17.20_1054.6311m/z	17.202093	1054.6311	0.018035218	1.5377666	0.6208365	up
17.20_824.5484m/z	17.202093	824.5484	0.020009123	2.0650053	1.0461454	up
17.21_263.1553m/z	17.209478	263.1553	0.006519921	1.6059104	-0.6833914	down
17.22_1030.6301m/z	17.219244	1030.6301	0.001703991	1.7671009	0.82138443	up
17.23_1171.2622m/z	17.226627	1171.2622	0.017391521	1.516339	0.60059226	up
17.29_303.2368m/z	17.287823	303.2368	5.59E-04	1.9443179	0.95926416	up
17.29_345.2479m/z	17.287823	345.24786	0.003810106	1.546448	0.6289583	up
17.29_466.3186n	17.287823	487.2932	2.06E-04	1.7445095	0.8028214	up
17.29_750.5494m/z	17.287823	750.54944	0.016415423	2.0176635	-1.0126855	down
17.29_776.5557m/z	17.287823	776.5557	0.005903326	1.8483397	0.8862299	up
17.32_873.5742m/z	17.322098	873.57416	0.02766816	1.6687335	-0.73875356	down
17.34_1032.6418m/z	17.339249	1032.6417	3.61E-04	1.5752171	0.6555507	up
17.34_279.2367m/z	17.339249	279.23672	5.91E-04	1.7730231	0.82621133	up
17.36_550.3076m/z	17.356384	550.30756	1.36E-04	1.8222393	0.8657124	up
17.38_807.0431m/z	17.37531	807.0431	0.023727434	1.5249475	0.6087596	up
17.39_600.3238m/z	17.39067	600.3238	0.01354588	1.6680671	-0.7381773	down
17.39_816.0630n	17.392462	781.0492	0.037267357	1.7230186	0.7849383	up
17.41_495.7495m/z	17.407818	495.74945	0.020129798	2.1426706	-1.09941	down
17.43_630.7098m/z	17.426756	630.7098	0.045600045	1.5193493	0.60345364	up
17.44_1198.7685m/z	17.44389	1198.7684	0.043987982	1.5747417	0.65511525	up
17.46_413.3273m/z	17.459244	413.32727	0.03573414	1.6740295	-0.74332494	down
17.51_1104.7524m/z	17.510675	1104.7524	0.03784041	1.7270608	0.7883189	up
17.51_856.5752m/z	17.512457	856.57526	0.018765409	1.5632755	0.644572	up
17.53_625.9177m/z	17.5296	625.9177	0.024000933	1.6277398	0.7028701	up
17.55_603.8950m/z	17.546873	603.895	0.009006283	1.5548614	0.636786	up
17.55_711.5276m/z	17.546873	711.5276	0.04101323	1.6366522	0.7107478	up
17.58_221.6202m/z	17.581154	221.62022	0.001616225	1.910084	0.93363607	up
17.62_1068.1843m/z	17.615423	1068.1843	0.01655883	1.8098183	0.85584486	up
17.62_1197.3038m/z	17.615423	1197.3038	0.049213454	1.5504004	0.63264084	up
17.62_2092.7769n	17.615423	705.9269	0.034186427	1.5434933	-0.6261992	down
17.62_704.4342m/z	17.615423	704.4342	0.029306116	1.5605829	0.64208496	up
17.62_822.0288n	17.615423	805.0255	0.028894989	1.5570345	0.6388009	up
17.63_534.3195m/z	17.630672	534.3195	7.10E-05	2.0940244	1.0662782	up
17.65_237.0831m/z	17.649725	237.08307	0.048316725	1.6462815	-0.71921104	down

17.70_365.2600m/z	17.701157	365.26	0.005454756	1.5056877	-0.5904226	down
17.73_826.5609m/z	17.733519	826.5609	0.009569609	2.066423	1.0471356	up
17.74_338.3397m/z	17.735456	338.33966	0.00992033	1.6463268	0.71925074	up
17.75_766.5424m/z	17.75067	766.54236	0.019493807	1.5761011	0.65636003	up
17.75_779.2876n	17.752579	762.28424	0.007310437	1.6298816	-0.70476717	down
17.77_766.2621n	17.769842	767.26935	0.014449995	1.7641479	-0.8189715	down
17.89_206.1254m/z	17.889847	206.12544	0.048966352	1.5388634	0.6218652	up
17.92_675.5378m/z	17.924133	675.53784	0.013840365	1.7963321	0.8450541	up
18.00_443.1999m/z	18.002096	443.19992	0.022189088	1.6449612	-0.7180536	down
18.04_188.5876m/z	18.038528	188.5876	0.015383177	1.5125028	0.5969378	up
18.10_1027.7711m/z	18.104961	1027.7711	0.015598347	1.5556755	-0.6375412	down
18.11_266.0782m/z	18.107117	266.0782	0.033717543	1.5710018	0.6516848	up
18.11_447.1974m/z	18.107117	447.19742	0.032331813	1.5507339	0.63295114	up
18.11_607.8897m/z	18.107117	607.8897	0.006932008	1.6357335	0.7099377	up
18.19_597.4167m/z	18.190672	597.41675	0.042454578	1.637902	0.71184903	up
18.21_311.3034m/z	18.210068	311.30338	0.02565091	2.3241692	-1.2167151	down
18.24_1167.5244m/z	18.244343	1167.5244	0.012458934	3.4197052	1.7738719	up
18.24_198.5459n	18.244343	439.12558	0.001332591	1.9098256	0.93344086	up
18.24_338.3397m/z	18.244343	338.3397	0.01583413	1.6423779	-0.71578604	down
18.24_339.3434m/z	18.244345	339.3434	0.015925562	1.6000828	-0.67814654	down
18.24_377.2236m/z	18.244343	377.22357	0.019889608	1.5202392	0.60429835	up
18.24_406.1684n	18.244343	407.17566	0.00145388	1.577001	0.6571835	up
18.24_623.2196m/z	18.244343	623.21954	0.004590423	2.415301	1.272203	up
18.26_1007.7351n	18.259232	988.7172	0.014104469	2.0314167	-1.0224862	down
18.26_1091.7214m/z	18.259232	1091.7214	0.04327728	1.515624	-0.5999119	down
18.26_1159.7089m/z	18.259232	1159.7089	0.043203637	1.5030584	-0.5879011	down
18.26_568.5950m/z	18.259232	568.59503	0.03528158	1.7196885	-0.7821473	down
18.29_575.1872m/z	18.293516	575.1872	0.04725245	1.567477	0.64844424	up
18.29_697.4493m/z	18.293516	697.4493	0.014692624	1.6492959	0.7218503	up
18.29_759.4169m/z	18.293516	759.41693	0.04513775	1.5696528	0.65044546	up
18.31_473.1529m/z	18.31293	473.15292	0.026679723	1.6492418	0.72180295	up
18.31_501.1221m/z	18.31293	501.12207	0.034155052	1.9465295	0.9609042	up
18.36_457.2675m/z	18.36436	457.2675	0.00430206	1.5979414	-0.6762145	down
18.40_312.3241m/z	18.398739	312.32413	0.043139745	1.6814443	-0.74970096	down
18.48_745.2986m/z	18.48444	745.2986	0.044200428	1.5279815	-0.6116271	down
18.48_812.2890m/z	18.48444	812.28906	0.003693058	1.5283422	-0.6119676	down
18.50_369.8860m/z	18.50159	369.886	2.87E-04	1.5238639	-0.6077341	down
18.50_544.8486m/z	18.50159	544.8486	0.007036872	1.5361984	-0.6193645	down
18.50_549.6684m/z	18.501593	549.6684	0.01296794	1.7493405	-0.80681115	down
18.50_935.1248n	18.50159	936.1321	0.016734658	1.6597395	-0.7309568	down
18.52_1011.1946n	18.518736	1053.2285	0.018031865	1.5378649	-0.62092876	down
18.54_1189.8256m/z	18.535887	1189.8257	0.004634207	2.001297	-1.0009353	down
18.54_1196.8420m/z	18.535887	1196.842	0.043522265	1.5202657	-0.6043235	down
18.55_1148.8077m/z	18.553038	1148.8077	0.013747873	1.896239	-0.9231408	down
18.55_1197.8352m/z	18.553038	1197.8352	0.002147926	1.6082361	-0.68547916	down
18.55_269.1111m/z	18.553038	269.1111	0.04887398	1.7551763	0.81161594	up
18.55_705.9556m/z	18.553038	705.95557	0.048509702	1.5724286	-0.6529945	down
18.64_705.1302m/z	18.638853	705.1302	0.04881026	1.524869	-0.60868526	down
18.69_757.5339n	18.687803	738.5161	0.017006744	1.5787288	0.65876335	up
18.72_834.1196n	18.72455	857.08655	0.045991927	1.5587664	-0.6404047	down
18.72_849.5734n	18.72209	870.5481	0.009487489	1.8545364	0.89105856	up
18.72_854.0504m/z	18.72455	854.0504	0.028109647	1.7500063	-0.8073601	down
18.79_1001.5157n	18.79065	1000.50836	0.024781633	1.8191069	0.86323035	up
18.79_721.4972m/z	18.787416	721.4972	0.020024607	1.5091532	-0.59373933	down

18.80_567.8687m/z	18.804567	567.8687	0.025399106	1.634298	-0.70867103	down
18.80_796.0590n	18.804567	797.0662	0.010102729	1.8775474	-0.9088493	down
18.81_804.5744m/z	18.8078	804.5744	0.015871027	1.6054237	0.6829541	up
18.82_1025.5438n	18.824936	1006.526	0.021550685	1.5483145	0.6306985	up
18.82_1068.4946m/z	18.824936	1068.4946	0.02673066	2.056641	1.04029	up
18.82_1074.5133m/z	18.824936	1074.5133	0.04091238	1.6821237	0.7502838	up
18.82_933.5288n	18.824936	932.5215	0.02281334	1.6546537	0.7265293	up
18.84_541.7153m/z	18.838987	541.7153	0.01363356	2.0182245	-1.0130867	down
18.84_668.6195m/z	18.838987	668.6195	0.007643411	1.5730487	-0.6535633	down
18.84_715.3782m/z	18.842087	715.37823	0.001703549	1.5388222	-0.6218265	down
18.84_957.5559n	18.842087	938.5381	0.01575046	1.6324606	0.7070482	up
18.84_959.6031m/z	18.838987	959.6031	0.003765622	1.6612451	-0.73226494	down
18.84_986.5969m/z	18.838987	986.5969	0.003069517	1.5890013	-0.66812027	down
18.91_369.1643m/z	18.907547	369.16428	0.019789355	2.1476078	1.1027305	up
18.92_632.9104n	18.924683	597.8965	0.0344868	1.6608722	-0.7319411	down
18.94_1156.3375m/z	18.941833	1156.3375	0.020794889	2.2885644	-1.1944429	down
18.96_648.9455m/z	18.958982	648.9455	0.03505074	1.6878176	-0.75515896	down
18.99_395.1099m/z	18.990658	395.10986	0.002595353	2.0397203	-1.0283713	down
19.01_265.1490m/z	19.007809	265.14905	0.02554543	1.5533458	-0.635379	down
19.01_333.1386m/z	19.007809	333.13864	0.021937937	1.6039795	-0.68165565	down
19.21_401.1585m/z	19.208971	401.15848	0.010642362	1.8225914	-0.8659912	down
19.21_537.3930m/z	19.211657	537.393	0.007343726	1.5155125	-0.5998057	down
19.26_463.7392m/z	19.263077	463.73917	0.032147795	1.5534552	-0.63548064	down
19.26_471.1045m/z	19.263077	471.1045	0.049987555	1.6191673	-0.69525206	down
19.28_307.1626m/z	19.277529	307.1626	0.009189721	2.1289303	-1.0901288	down
19.28_868.0919m/z	19.280228	868.0919	0.032267865	1.5453644	-0.62794703	down
19.31_1007.5005n	19.314516	972.48663	0.007183861	1.9877077	-0.9911056	down
19.35_1000.5101m/z	19.34881	1000.5101	0.026381325	1.7028009	-0.7679097	down
19.35_1163.6837m/z	19.34881	1163.6837	0.02374098	2.1201072	-1.0841372	down
19.35_995.4755n	19.34881	996.4827	0.023018751	1.8798202	-0.9105947	down
19.38_1149.6434m/z	19.38039	1149.6433	0.047908396	1.7538527	-0.8105276	down
19.42_1151.3668m/z	19.41736	1151.3668	0.006738042	2.1913471	-1.131818	down
19.43_206.1648n	19.434626	207.17204	0.045581844	1.6301081	0.7049676	up
19.45_1139.7730m/z	19.451777	1139.773	0.024351612	1.5856298	-0.665056	down
19.45_218.5758m/z	19.451777	218.57584	0.023378428	2.1023138	1.071978	up
19.45_275.0792m/z	19.451777	275.0792	0.012072762	1.5951664	0.67370695	up
19.45_328.2247m/z	19.451777	328.22467	0.031007402	1.7070283	0.77148694	up
19.45_486.1507m/z	19.451777	486.15076	0.04960649	1.9841218	0.9885006	up
19.45_572.8576m/z	19.451777	572.8576	0.041560568	1.6348008	0.70911485	up
19.47_1187.8462m/z	19.46889	1187.8462	0.03370927	1.6446414	-0.717773	down
19.47_1191.8379m/z	19.46889	1191.8379	0.006473437	1.7299249	-0.79070944	down
19.47_307.7137m/z	19.468891	307.71365	0.024056133	1.6683861	0.7384532	up
19.47_336.2187m/z	19.46889	336.21872	0.031121302	1.8469414	0.88513803	up
19.47_393.2178m/z	19.46889	393.2178	0.017506853	1.8912548	0.9193437	up
19.47_783.0629m/z	19.46889	783.06287	0.007820418	2.0882106	-1.0622672	down
19.50_328.3184m/z	19.503178	328.31836	0.007052444	3.0262184	1.5975161	up
19.50_580.8555m/z	19.503178	580.8555	0.012340942	1.8348452	0.87565833	up
19.52_603.8514m/z	19.520329	603.85144	0.03066939	1.5697569	-0.6505411	down
19.64_640.9585m/z	19.640324	640.9585	0.0275948	1.7583982	-0.8142618	down
19.64_667.9678m/z	19.640324	667.96783	0.03047024	1.5727942	-0.6533299	down
19.66_1139.7762m/z	19.657576	1139.7762	0.04874318	1.5773301	-0.6574846	down
19.69_504.8924m/z	19.686155	504.89243	0.03995426	2.3422077	-1.227869	down
19.69_586.8641m/z	19.686155	586.8641	0.012067184	1.5822048	0.66193634	up
19.70_1094.7105m/z	19.703306	1094.7106	0.03291838	1.8608452	-0.89595807	down

19.70_1162.3732m/z	19.703306	1162.3732	0.014148318	1.887739	-0.91665924	down
19.70_1165.8086m/z	19.703306	1165.8086	2.06E-04	2.289915	-1.1952941	down
19.70_1166.8022m/z	19.703306	1166.8021	0.005283736	1.7526703	-0.80955464	down
19.70_1188.8474m/z	19.703306	1188.8474	0.038875647	1.5664496	-0.64749837	down
19.70_705.9932m/z	19.703306	705.9932	0.04838672	1.9394811	0.9556707	up
19.72_1077.7450n	19.720444	1078.7523	0.021713763	1.6398233	-0.7135404	down
19.72_1149.8104m/z	19.720444	1149.8104	0.005106756	1.7373867	-0.79691887	down
19.72_1164.3558m/z	19.720444	1164.3557	0.004244496	2.090376	-1.0637624	down
19.72_801.6691m/z	19.720444	801.6691	0.025746208	1.5559471	0.63779294	up
19.72_813.6730m/z	19.720442	813.67303	0.030120747	1.5237108	0.6075891	up
19.72_949.6051m/z	19.720444	949.6051	0.001119191	1.6130213	-0.68976545	down
19.72_960.6577m/z	19.720444	960.6577	0.013760105	1.5419711	-0.6247757	down
19.72_981.6662m/z	19.720444	981.66614	0.04494337	1.6077334	-0.68502814	down
19.81_465.3121m/z	19.808956	465.31213	0.03445318	1.6291856	0.7041509	up
19.83_762.5156m/z	19.826107	762.51556	0.022530861	1.6376882	0.7116607	up
19.96_307.7068m/z	19.960556	307.7068	0.04723618	1.5620776	-0.6434662	down
19.99_410.7577m/z	19.994825	410.75772	0.00598081	1.633424	0.70789933	up
2.00_169.1643m/z	1.9969002	169.16428	0.018048665	1.7029884	0.7680686	up
2.44_252.0909m/z	2.442383	252.0909	0.043501526	1.5978954	-0.676173	down
2.44_342.1220m/z	2.442383	342.12198	0.013523366	1.8359135	-0.8764981	down
2.44_402.0785m/z	2.442383	402.07846	0.002315852	1.9654474	-0.97485775	down
2.46_400.0822m/z	2.459518	400.0822	0.028707381	1.6492099	-0.721775	down
2.46_410.1100m/z	2.459518	410.11	0.007604873	1.8654987	-0.8995613	down
2.63_270.0987m/z	2.6309507	270.0987	0.00800997	1.7436893	-0.802143	down
2.63_376.1018m/z	2.6309507	376.10175	0.010254408	1.7389202	-0.7981917	down
2.63_428.1186m/z	2.6309507	428.11862	0.004177824	1.7101542	-0.7741264	down
2.63_659.1298m/z	2.6309507	659.1298	0.006442026	1.7452257	-0.80341357	down
2.65_247.0439m/z	2.6480846	247.0439	4.88E-05	2.9993691	-1.5846591	down
2.68_130.0857m/z	2.682366	130.08574	0.004606862	1.628603	-0.7036349	down
2.68_293.1487n	2.682366	292.14145	0.007287748	1.7056208	-0.7702969	down
2.68_329.1267n	2.682366	350.10138	0.00394865	1.6662	-0.7365616	down
2.68_331.1252n	2.682366	352.09982	0.004561484	1.7390279	-0.798281	down
2.68_360.1298m/z	2.682366	360.12976	0.006657583	1.6585094	-0.7298871	down
2.68_422.1020m/z	2.682366	422.10202	0.010225421	1.7039802	-0.76890856	down
2.68_480.1438m/z	2.682366	480.14377	0.038290817	1.6473699	-0.7201645	down
2.70_202.1093m/z	2.6995165	202.10928	0.006761856	1.7270211	-0.7882857	down
2.70_244.1219m/z	2.6995165	244.12195	0.004386674	1.7710193	-0.82457995	down
2.70_286.0642m/z	2.6995165	286.06424	0.04420568	1.6932672	-0.7598097	down
2.70_418.1715m/z	2.6995165	418.17154	0.01658567	1.6664926	-0.73681486	down
2.70_486.1604m/z	2.6995165	486.1604	0.006709321	1.7675397	-0.82174265	down
2.70_586.2966n	2.6995165	585.2893	0.00471891	2.2505817	-1.170298	down
2.70_665.2317m/z	2.6995165	665.23175	0.007987618	1.948897	-0.9626578	down
2.72_476.1332m/z	2.7166514	476.13324	0.001184881	1.8459605	-0.8843717	down
2.75_455.2010n	2.745451	456.2083	0.031943005	1.7093006	-0.77340615	down
2.80_380.0988m/z	2.8023674	380.09885	1.09E-04	2.6057134	-1.3816783	down
2.85_228.0346m/z	2.8537996	228.03458	0.00175886	2.5443811	-1.3473148	down
2.85_249.0539m/z	2.848318	249.05386	0.004181213	1.6978347	-0.763696	down
3.22_315.1917m/z	3.2197504	315.1917	0.008641013	1.946545	-0.9609157	down
3.40_513.1884m/z	3.3966508	513.1884	0.007258975	1.7201211	-0.78251016	down
3.43_236.0951m/z	3.4309337	236.0951	0.007019697	1.6513504	-0.7236462	down
3.43_327.1333n	3.4309337	326.12607	0.006601415	1.6744571	-0.74369335	down
3.43_363.1120n	3.4309337	384.0867	0.004217519	1.7723732	-0.8256824	down
3.43_394.1145m/z	3.4309337	394.11453	0.004039166	1.6914251	-0.75823927	down
3.43_410.0857m/z	3.4309337	410.08566	0.006721542	1.739947	-0.7990433	down

3.43_438.1420m/z	3.4309335	438.14203	0.031256817	1.6543909	-0.7263002	down
3.43_462.1029m/z	3.4309337	462.10287	0.001797359	1.8666455	-0.9004479	down
3.43_654.2671n	3.4309335	653.25977	0.026302328	1.7826931	-0.8340584	down
3.45_217.0306m/z	3.4480658	217.03058	2.30E-04	2.4804153	1.3105817	up
3.45_239.0135m/z	3.4480658	239.01349	1.42E-04	2.7568254	1.4630079	up
3.52_323.0415m/z	3.5166326	323.0415	0.040874436	1.7289021	0.7898562	up
3.67_256.1292m/z	3.6654506	256.12924	0.04005144	1.5833503	-0.66298044	down
4.00_218.0383n	3.9966166	217.03102	5.97E-04	2.4053323	1.2662362	up
4.08_416.0977m/z	4.076631	416.09766	5.98E-06	2.1564853	-1.1086819	down
4.09_323.0414m/z	4.0937657	323.04138	0.003501365	2.2296894	1.1568427	up
4.14_438.1977m/z	4.1397347	438.1977	0.004976601	1.5982383	-0.67648256	down
4.16_456.2083m/z	4.1568656	456.2083	0.001171011	1.7388389	-0.79812425	down
4.19_114.0667m/z	4.1911483	114.0667	0.046039097	1.5991646	-0.67731845	down
4.40_173.0382m/z	4.3968663	173.0382	0.041621197	1.628064	0.7031574	up
4.41_256.9934m/z	4.414001	256.9934	0.014640258	2.068999	1.048933	up
4.45_149.5124m/z	4.448284	149.51244	0.007363007	1.7925082	0.84197974	up
4.63_496.1045n	4.625183	495.09726	0.048860814	1.58978	-0.6688271	down
4.66_573.1726m/z	4.659468	573.17255	0.007890629	1.8692504	-0.90245986	down
4.69_257.1034m/z	4.693752	257.10336	0.010381728	1.6227186	-0.69841284	down
4.71_324.0765m/z	4.7108808	324.07648	0.009905502	1.5028952	-0.5877444	down
4.75_173.0404m/z	4.745182	173.0404	4.62E-04	2.4754574	1.3076952	up
4.75_399.1153m/z	4.745182	399.1153	0.007151401	2.0024865	1.0017924	up
4.75_713.1899m/z	4.745182	713.1899	4.54E-04	1.5114566	-0.5959396	down
4.76_241.0315m/z	4.762315	241.03156	0.010624834	2.0082293	1.005924	up
4.81_406.1354m/z	4.8137507	406.1354	0.038738627	1.6149528	-0.691492	down
4.83_179.1888m/z	4.830886	179.18883	0.037107203	1.6584555	-0.7298403	down
4.83_240.0904m/z	4.830886	240.09044	0.033202693	1.837562	-0.8777929	down
4.83_449.0956m/z	4.830886	449.0956	8.54E-04	1.7859513	-0.8366927	down
4.94_398.0811n	4.9397163	421.07037	0.04520918	1.7239206	-0.7856933	down
5.23_375.1874m/z	5.231136	375.1874	0.025109237	1.5224904	-0.6064331	down
5.31_421.1861m/z	5.3051643	421.18613	0.003942566	1.6059674	-0.6834426	down
5.31_467.1103m/z	5.305165	467.11035	1.56E-05	2.0628235	-1.0446204	down
5.44_446.9971m/z	5.4423027	446.99707	0.03377818	1.7639273	-0.81879115	down
5.77_178.0008m/z	5.7740016	178.00081	0.004368935	1.6939617	0.7604013	up
5.77_233.0966m/z	5.7740016	233.09662	5.23E-06	2.0149727	-1.0107603	down
5.80_437.0908m/z	5.8023033	437.09076	5.67E-04	1.9248943	-0.9447792	down
6.02_256.0679m/z	6.019436	256.0679	0.016914412	2.2327628	-1.1588299	down
6.09_290.0678m/z	6.0879984	290.06775	0.006190945	1.5719658	-0.65256983	down
6.68_348.1897m/z	6.6768503	348.18973	0.021746377	2.525283	-1.3364451	down
7.09_230.9985m/z	7.0937004	230.99847	0.03976843	1.5663294	0.6473876	up
8.07_498.1574n	8.065097	497.15015	0.003774122	1.546287	-0.6288081	down
9.07_237.0782m/z	9.0708	237.07823	0.002910139	2.4322584	1.2822964	up
9.40_358.0541m/z	9.396494	358.0541	0.004395961	1.881724	-0.912055	down
9.52_669.0822m/z	9.516816	669.08215	0.033029076	1.763549	-0.8184816	down
9.65_315.1030m/z	9.653628	315.10303	0.017834058	1.5427657	0.625519	up
9.80_197.0434m/z	9.802529	197.04338	0.038995784	1.578845	-0.65886956	down

Supplementary Table 2: Exogenous and enogenous metabolites. Paired t.test, no correction p-value ≤ 0.05, FC 1.5.

Compound	Retention time (min)	Mass	Accepted Compound ID	Accepted Description	p ([Post] Vs [Pre])	FC (abs) ([Post] Vs [Pre])	Log FC ([Post] Vs [Pre])	Regulation ([Post] Vs [Pre])	exogenous
0.76_509.8870n	0.7567	508.8796762	HMDB0246058	3,5-Diiodothyropropioni	0.016754359	1.8940355	0.9214633	up	x
0.79_448.8568m/z	0.790983333	448.8567865	HMDB0244565	Tris(1,3-dichloro-2-propy	0.037312448	1.9533483	-0.9659492	down	x
0.79_554.9806m/z	0.790983333	554.9806073	HMDB0248266	1-(2,4-Dichlorophenyl)-5	0.011696158	2.772687	1.4712847	up	x
0.79_877.9494m/z	0.790983333	877.9493894	HMDB0248429	Angiotensinogen	0.044428974	1.6447862	0.7179001	up	
0.80_353.1303m/z	0.802433333	353.1302543	HMDB0062186	1-Deoxy-1-(N6-lysino)-D	4.73E-04	1.5791446	-0.65914327	down	x
0.81_308.0646n	0.808116667	329.0392927	CSID24846246	2-(1,3-Benzodioxol-5-yl)	0.015644401	2.6603048	1.4115915	up	x
0.82_331.1472m/z	0.819566667	331.1471939	HMDB0062186	1-Deoxy-1-(N6-lysino)-D	0.006642255	1.6076539	-0.6849568	down	x
0.83_695.7793m/z	0.825266667	695.7792877	HMDB0256285	Pentetoreotide	0.004418047	1.625648	-0.70101494	down	x
0.83_854.4030m/z	0.825266667	854.4029707	HMDB0116068	CDP-DG(a-13:0/i-12:0)	0.023055285	1.7101332	0.7741087	up	
0.84_250.8688m/z	0.836716667	250.8687754	HMDB0032079	2,4-Dibromophenol	0.03756708	2.294894	1.1984274	up	x
0.88_846.1632m/z	0.8767	846.1631912	HMDB0300835	4-Methylpentanoyl-CoA	0.020493709	1.7327727	-0.7930824	down	
0.88_964.7675m/z	0.8767	964.7675096	HMDB0114629	PE-NMe2(24:0/24:0)	0.037907135	1.5166286	-0.60086787	down	
0.89_250.0969m/z	0.893833333	250.0969237	HMDB0001983	5'-Deoxyadenosine	0.015466255	1.7051629	-0.76990956	down	
0.89_257.0583m/z	0.893833333	257.0582979	HMDB0029045	Serylmethionine	0.014568478	1.6206636	-0.6965847	down	
0.89_365.0767m/z	0.893833333	365.0766821	HMDB0259961	Z-Ala-ONp	0.011496306	1.7361885	-0.7959236	down	x
0.89_367.0738m/z	0.893833333	367.0737972	CSID24843012	3,9,10-Trimethoxy-6a,11	0.007789504	2.280611	-1.1894203	down	x
0.89_388.9446m/z	0.893833333	388.9445874	HMDB0062643	5-O-phosphonato-alpha-	0.012042287	2.1803746	-1.124576	down	
0.91_160.0969m/z	0.905366667	160.0968871	HMDB0011757	N-Acetylvaline	0.0245198	1.6515797	0.7238466	up	
0.91_282.0698m/z	0.910983333	282.0698475	HMDB0029144	gamma-Glutamylasparag	0.009010732	1.8943775	-0.92172384	down	x
0.91_338.0742m/z	0.910983333	338.0741518	HMDB0061168	N2-Monodes-methylniza	0.004052527	1.6508809	-0.7232361	down	x
0.91_947.0894m/z	0.910983333	947.0893547	HMDB0304165	4-(2'-carboxyphenyl)-4-c	0.0012865	1.6341016	-0.7084977	down	x
0.93_243.0656m/z	0.928116667	243.0655698	HMDB0032838	Dorsteniol	0.04233706	1.5268319	0.61054116	up	x
0.95_207.0535m/z	0.945266667	207.0535247	HMDB0304400	L-arogenate	0.001409817	1.722385	-0.7844077	down	x
0.95_267.0751m/z	0.945266667	267.0751432	HMDB0013713	N-Acetyltryptophan	0.011192644	1.7230735	-0.78498423	down	
0.95_305.1024m/z	0.945266667	305.1024341	CSID24608524	7-oxocurvularin	0.04416314	1.6279148	-0.70302516	down	x
0.98_188.0949m/z	0.97955	188.0948696	HMDB0304554	DIMBOA trihexose	0.02957895	1.5344837	-0.61775327	down	x
0.98_486.9887m/z	0.97955	486.9886864	HMDB0248355	Phosphoaminophosphor	0.030196983	2.0333674	-1.023871	down	x
1.00_111.0066m/z	0.996683333	111.0065703	HMDB0001051	Glyceraldehyde	0.03169592	2.3296943	1.2201406	up	
1.00_175.0267m/z	0.996683333	175.0267247	CSID389014	D-glucurono-6,2-lactone	0.001579856	1.9681259	-0.97682256	down	x

1.00_446.1562m/z	0.996683333	446.1562074	HMDB0254392	Mecinarone	0.020330878	1.5241299	-0.60798585 down	x
1.11_489.1014m/z	1.110983333	489.1014144	HMDB0041746	Irisolidone 7-O-glucuron	0.020047422	1.5976063	-0.6759119 down	x
1.23_622.9607m/z	1.23115	622.9606736	HMDB0255059	N-Acetyl-L-phenylalanyl-	0.04752368	1.7044762	-0.7693285 down	x
1.32_243.0654m/z	1.316683333	243.0653731	HMDB0028752	Aspartyl-Glutamate	0.040647753	1.544766	-0.62738824 down	x
1.32_278.1273m/z	1.316683333	278.1272509	HMDB0252498	Fructosylvaline	0.003583125	1.9619426	-0.97228277 down	x
1.33_116.0705m/z	1.333833333	116.0705203	HMDB0000883	L-Valine	0.013315325	1.8026835	-0.8501461 down	
1.33_188.0946m/z	1.333833333	188.0945874	HMDB0062181	N-Lactoylvaline	0.004938704	1.8232882	-0.86654264 down	
1.33_307.0377m/z	1.333833333	307.0377421	CSID58574	Deoxyuridine monophos	0.028361771	1.6610477	-0.7320935 down	
1.33_365.0372m/z	1.333833333	365.0372061	HMDB0304274	betanidin quinone	0.009093703	1.8920474	-0.9199482 down	x
1.33_447.1633n	1.333833333	468.1379995	HMDB0252503	Methyl (2S)-2-[[4-[[(2R)-:	0.015473031	1.903036	-0.9283028 down	x
1.33_508.0585m/z	1.333833333	508.0585216	HMDB0006744	3-Carboxy-1-hydroxypro	0.011763437	1.9478915	-0.9619133 down	
1.33_637.1682n	1.333833333	636.16097	CSID115268918	5-[(6-O-Acetyl-beta-D-gl	0.007988762	2.3406994	-1.2269397 down	x
1.35_201.0393m/z	1.350983333	201.0392679	HMDB0035149	Chrycorin	0.011626238	1.5699596	-0.65072745 down	x
1.42_361.1960m/z	1.41975	361.1959818	HMDB0003933	Pentosidine	0.04367991	1.7133973	-0.7768597 down	
1.44_290.0912m/z	1.436683333	290.0911958	HMDB0000230	N-Acetylneuraminic acid	5.06E-04	1.9973924	-0.9981178 down	
1.51_636.1566m/z	1.50525	636.1566349	HMDB0248376	Anacetrapib	7.72E-04	3.065407	-1.6160786 down	x
1.57_128.0337m/z	1.573833333	128.0336871	HMDB0000148	L-Glutamic acid	0.02684131	1.7142011	-0.77753633 down	
1.57_185.9950m/z	1.573833333	185.9950147	CSID8468	aminitrozole	0.047704514	1.6237485	-0.69932824 down	x
1.57_240.0513m/z	1.573833333	240.0513494	HMDB0255868	O-Succinyl-L-homoserine	0.01261215	2.1600568	-1.1110692 down	x
1.57_337.0290m/z	1.573833333	337.0289528	CSID4445016	TAMARIXETIN	0.031213025	1.7404081	-0.7994256 down	x
1.57_339.0142m/z	1.573833333	339.014173	HMDB0037357	3,3',4',5,5',8-Hexahydrox	0.001453779	1.8433726	-0.8823477 down	x
1.57_342.0435m/z	1.573833333	342.0435302	CSID19992713	N-[3-Carboxy-2-(carboxy	0.020188767	2.2722666	-1.1841321 down	
1.59_346.0452m/z	1.590966667	346.0452002	HMDB0253713	isoxazolyl penicillin	0.04056459	2.2478454	-1.1685429 down	x
1.64_196.0249m/z	1.6424	196.024876	CSID88007	N-Acetylaspartic acid	0.023533659	1.5408987	-0.62377197 down	
1.73_342.0446m/z	1.7281	342.0446206	HMDB0013220	Beta-Citryl-L-glutamic ac	0.009920922	2.0599792	-1.0426297 down	
10.01_439.1648m/z	10.00791667	439.1647578	HMDB0302053	Cichorioside G	0.026315639	1.699408	-0.7650323 down	x
10.20_413.1671m/z	10.19648333	413.1670662	HMDB0259867	Vofopitant	0.035825487	1.7455391	-0.8036726 down	x
10.59_400.1988m/z	10.59076667	400.1987992	HMDB0250692	4-Isopropylphenserine	0.0111428	1.5490886	0.63141966 up	x
10.66_452.1967m/z	10.65933333	452.196666	HMDB0304857	Tirbanibulin	0.00894521	1.5597111	-0.6412788 down	x
10.69_503.2624m/z	10.68825	503.2624468	HMDB0011875	Ganglioside GM1 (d18:0,	0.044839337	1.5775892	-0.6577215 down	
10.78_210.0797m/z	10.77933333	210.0797039	CSID4444887	Enicoflavine	0.049059168	1.5434341	-0.62614393 down	x
10.83_462.1792m/z	10.83075	462.1791568	HMDB0061137	Dihydroisomorphine-6-g	8.70E-04	1.6009436	-0.6789224 down	x
10.83_530.1678m/z	10.83075	530.1677898	HMDB0029361	Alkaloid RC	3.84E-05	1.833809	-0.87484336 down	x

11.10_414.3364m/z	11.09966667	414.3363998 HMDB0062342	N-Stearoyl phenylalanine	0.014545579	2.0332472	-1.0237856 down	
11.12_505.1507m/z	11.11646667	505.1507155 HMDB0035733	Dukunolide E	0.011022789	1.6299536	0.7048309 up	x
11.29_227.0406m/z	11.2879	227.0406153 HMDB0247589	9-Deaza-9-(3-thienylmet	0.016012695	1.7454605	-0.8036077 down	x
11.41_366.1045m/z	11.4079	366.1045222 HMDB0038952	Niazicin	0.010224287	2.0664306	-1.0471408 down	x
11.41_421.0628m/z	11.4079	421.062778 HMDB0259180	TRICIRIBINE PHOSPHATE	0.043698255	1.8805646	-0.91116583 down	x
11.46_255.1187m/z	11.45931667	255.1187218 HMDB0038319	Decarbamoylsaxitoxin	3.81E-04	1.7439003	0.80231756 up	x
11.53_597.2396m/z	11.52788333	597.23962 HMDB0260020	5,10,15,20-Tetraphenyl-	0.04434461	1.5994982	-0.67761934 down	x
11.63_405.1218m/z	11.63075	405.1218236 HMDB0060923	Naproxen O-glucuronide	0.034428537	2.749567	-1.4592044 down	x
11.73_337.1461m/z	11.7336	337.1461057 CSID4476408	8-geranyloxypsoralen	0.0062594	1.7255516	-0.78705764 down	x
11.91_229.0553m/z	11.90501667	229.0552684 HMDB0304052	2-(5'-methylthio)pentyln	0.04773376	1.5931745	-0.67190427 down	x
12.43_209.0878m/z	12.43073333	209.0877867 CSID120970	Alanylvaline	0.022987157	1.7256304	0.7871235 up	
12.43_399.1104m/z	12.43073333	399.1104194 CSID24845785	8-Hydroxy-3,5,7,3',4',5'-l	0.030210761	1.5644299	-0.645637 down	x
12.47_357.1017m/z	12.46501667	357.1017403 HMDB0253945	Lactobionic acid	0.014431571	1.5794089	-0.6593847 down	x
12.47_437.0560m/z	12.46501667	437.0559544 CSID24844723	3,5-Dihydroxy-2-(4-hydr	0.007247953	1.9360402	-0.95310885 down	x
12.47_506.0523n	12.46501667	505.0450534 HMDB0015199	Cefditoren	0.038703278	1.8293104	-0.8712999 down	x
12.49_429.1149m/z	12.49405	429.1148856 CSID23327231	Physcion 8-glucoside	0.013680263	1.8826052	-0.91273046 down	x
12.62_461.2937m/z	12.6193	461.2937321 CSID390406	Ajugasterone C	1.42E-04	1.8414747	0.8808615 up	x
12.64_252.0762m/z	12.63645	252.0762083 HMDB0255759	Novuridine	0.013621724	1.8224154	0.8658518 up	x
12.69_338.1516n	12.68786667	337.1443221 CSID4581520	Bergamottin	1.98E-04	1.5397713	-0.62271607 down	x
12.71_451.0743m/z	12.70501667	451.074253 HMDB0302247	Lirodenine	0.005072738	2.177781	-1.1228589 down	x
12.84_372.1379m/z	12.84215	372.137876 HMDB0253369	Icotinib	0.004224652	1.6618304	-0.7327732 down	x
13.04_358.1709m/z	13.04215	358.1709146 CSID74853856	N-[2-(3,4-Dimethoxyphe	0.004394331	1.8362517	-0.8767638 down	x
13.06_613.3627m/z	13.0593	613.3626674 CSID4444753	Geissospermine	0.006077101	2.492913	1.3178325 up	x
13.09_680.3454n	13.09358333	701.3200798 HMDB0035433	Canarigenin 3-[glucosyl-(	0.018106526	2.0513787	-1.0365938 down	x
13.37_304.0391m/z	13.36786667	304.0391131 CSID5901	cytidine 5,Ä≤-monophos	0.029593918	1.7317793	-0.7922551 down	
13.39_429.2015m/z	13.385	429.2015059 HMDB0114745	LysoPA(18:4(6Z,9Z,12Z,1	0.005314179	1.5125154	-0.5969499 down	
13.39_515.2888m/z	13.385	515.2888405 CSID391741	3-O-(alpha-L-olivosyl)ole	0.011773074	1.6023202	-0.6801625 down	x
13.45_507.2277m/z	13.45358333	507.2276826 HMDB0038611	Gibberellin A37 glucosyl	0.03671311	1.973636	-0.98085594 down	x
13.48_394.2754m/z	13.4827	394.2753602 HMDB0242022	N-Docosahexaenoyl Thre	0.046887442	1.6711013	0.7407992 up	
13.54_528.1604m/z	13.53928333	528.1603766 HMDB0251704	Edoxaban	0.012273391	2.3353496	-1.2236385 down	x
13.56_321.1404m/z	13.55643333	321.1403541 HMDB0254504	Metaphit	0.013511065	1.7795403	-0.8315046 down	x
13.59_459.1821m/z	13.59071667	459.1821373 HMDB0040613	Kanzonol G	0.029501164	1.8413815	-0.88078856 down	x
13.61_675.3271m/z	13.60785	675.3271219 CSID17220943	(2R,2'R,3S,3'R)-2,2',3-Tri	0.006813169	1.5390111	-0.6220037 down	x

13.63_457.1573m/z	13.625	457.1573392	HMDB0015013	Flupenthixol	0.003915267	1.5377175	-0.6207904 down	x
13.64_443.1582m/z	13.64215	443.1581703	HMDB0013946	8-Hydroxycarvedilol	0.002465464	1.6541858	-0.72612125 down	x
13.78_319.1352m/z	13.77928333	319.135243	HMDB0029514	Licochalcone A	3.67E-05	1.563302	-0.6445965 down	x
13.79_309.1311m/z	13.7856	309.1311062	HMDB0000050	Adenosine	0.015093282	1.5221747	0.60613394 up	
13.80_318.2999m/z	13.80275	318.2999231	HMDB0004610	Phytosphingosine	5.11E-04	3.2768807	1.7123232 up	
13.82_375.0744m/z	13.81988333	375.0743838	HMDB0255282	N(4)-Acetylsulfadimetho	0.031694513	2.1199567	-1.0840348 down	x
13.83_497.2777m/z	13.83071667	497.2777408	HMDB0255480	12-[Methyl-(4-nitro-2,1,3	0.004069313	1.532954	-0.61631435 down	x
13.85_384.3230m/z	13.85418333	384.3230184	HMDB0030385	2,4,8-Eicosatrienoic acid	0.006903545	1.7044718	0.7693247 up	x
13.95_329.2366m/z	13.9507	329.236598	HMDB0302836	Sativic acid	0.033029057	1.6015576	0.67947567 up	x
14.17_273.0691m/z	14.16785	273.0690565	HMDB0038850	Cycasin	0.003168749	1.589615	0.6686774 up	x
14.17_406.1375m/z	14.16785	406.1375341	HMDB0254740	Minoxidil Glucuronide	0.040260307	2.4896703	-1.3159547 down	x
14.19_432.1503m/z	14.185	432.150302	HMDB0258533	Succinyl-trialanine-4-nitr	0.006918493	1.522255	0.60621 up	x
14.26_239.0793m/z	14.25988333	239.0793196	HMDB0248863	Bapta	0.010256497	2.0383556	1.0274057 up	x
14.29_226.9484m/z	14.29416667	226.9484304	HMDB0060363	2,5-Dichloro-4-oxohex-2	0.018285286	1.7951835	0.84413135 up	x
14.34_321.1498m/z	14.33928333	321.1498352	HMDB0000010	2-Methoxyestrone	8.37E-05	1.5384978	-0.62152237 down	
14.35_358.9795m/z	14.3457	358.9794655	HMDB0247537	Fluorapacin	0.017810613	2.1454244	1.101263 up	x
14.37_460.2948m/z	14.37356667	460.294833	CSID24607453	7-{(3S,4S,5R)-4-[(1E)-3-H	0.004327671	1.8566513	-0.8927029 down	
14.37_786.3977m/z	14.37356667	786.3976542	CSID21258204	Cethromycin	0.006659401	1.5210036	-0.60502356 down	x
14.39_429.2182m/z	14.3907	429.2181581	HMDB0029347	Acuminoside	5.50E-04	1.5283737	-0.61199737 down	x
14.39_499.2919m/z	14.3907	499.291854	HMDB0014554	Mupirocin	0.001118688	1.5272464	-0.61093277 down	x
14.41_633.2566m/z	14.40785	633.256587	HMDB0039121	Crocin 3	7.34E-04	1.61807	-0.694274 down	x
14.45_386.3094m/z	14.44855	386.3093582	HMDB0062339	N-Palmitoyl phenylalanir	0.045934897	1.8626549	-0.8973604 down	
14.49_408.1709m/z	14.49355	408.1709346	HMDB0259296	Tropantiol	8.41E-04	1.66203	-0.7329464 down	x
14.51_559.1171m/z	14.5107	559.1170855	HMDB0256077	Paeoniflorin sulfonate	0.003329226	1.5500169	-0.6322839 down	x
14.53_290.1694n	14.53426667	255.1555463	HMDB0244757	16alpha-Fluoroestradiol	0.046120957	1.5103858	0.59491706 up	x
14.53_435.2732m/z	14.52785	435.2731749	HMDB0296902	DG(20:4(5Z,7E,11Z,14Z)-	5.66E-04	1.6587929	-0.7301337 down	
14.56_253.1476m/z	14.56213333	253.1475528	HMDB0241923	N-Palmitoyl Cysteine	5.67E-04	1.512098	-0.5965516 down	
14.58_509.2363n	14.57926667	508.2290402	HMDB0254746	Miproxifene phosphate	0.021347899	1.7573303	-0.81338537 down	x
14.65_375.1880m/z	14.64783333	375.1880013	HMDB0256037	p-Fluorofentanyl	0.003978572	1.6744924	-0.7437238 down	x
14.66_321.1496m/z	14.66498333	321.1495623	HMDB0006772	Adrenosterone	5.72E-06	1.6040126	-0.68168545 down	
14.78_385.2082m/z	14.78498333	385.2082395	CSID2252	benzquinamide	0.005845116	1.5330954	-0.61644745 down	x
14.82_731.3701m/z	14.81926667	731.3700663	CSID113380954	(2S,8R,19E)-1,2,5,18-Tet	0.013894185	1.6032625	-0.68101066 down	x
14.83_268.2634m/z	14.82583333	268.2633671	HMDB0031081	2-Pentadecanone	0.03036803	1.9327018	0.95061904 up	x

14.84_295.1571m/z	14.83641667	295.1571113	CSID13628077	3-Hydroxytetradecanedi	9.76E-05	1.6547801	-0.72663957	down	
14.91_170.0956m/z	14.91155	170.0955711	CSID81836	N-Methyl-L-histidine	0.04409952	1.5751343	0.65547484	up	
14.91_205.1479m/z	14.91155	205.1478819	HMDB0015557	Pheniramine	0.04354215	2.1384678	1.0965774	up	x
14.91_286.3087m/z	14.91155	286.3087322	HMDB0258433	Spisulosine	0.024991505	2.8673744	1.5197303	up	x
14.96_521.2489m/z	14.9564	521.2489257	HMDB0248584	Arginine-glycine-asparta	0.04916055	1.5122046	-0.5966534	down	x
14.96_539.2901m/z	14.9564	539.2901006	CSID72391199	3,7,15-Trihydroxy-11,23-	6.19E-04	1.9743012	-0.9813421	down	x
14.96_590.3112n	14.9564	589.3039231	HMDB0004158	Urobilinogen	0.007035755	1.5572509	-0.63900137	down	
14.96_616.2580n	14.9564	615.2507258	HMDB0249696	Casopitant	4.18E-04	1.8491118	-0.8868324	down	x
14.97_283.1551m/z	14.97355	283.1551425	CSID74854303	L-Glutaminyl-L-Arginine	6.47E-04	1.7100081	-0.7740032	down	
14.97_324.1745n	14.97355	323.1672639	HMDB0255721	Tecastemizole	4.89E-06	1.6877272	-0.7550817	down	x
15.00_445.2493m/z	15.00213333	445.2493131	CSID25937160	3Œ±,7Œ±,12Œ±,16Œ±-t	0.004847816	1.6763755	-0.74534535	down	
15.00_537.2599m/z	15.00213333	537.2599387	CSID391741	3-O-(alpha-L-olivosyl)ole	0.028688455	1.5049524	-0.58971786	down	x
15.02_558.2471n	15.01926667	557.239831	HMDB0005006	Atorvastatin	0.00118408	1.72882	-0.78978765	down	x
15.02_574.2148n	15.01926667	573.2075643	HMDB0006825	Tetrahydrofolyl-[Glu](2)	0.003804523	1.8973418	-0.92397964	down	
15.02_601.2080m/z	15.01926667	601.2080093	HMDB0304275	biliverdin-IX-alpha	0.00370947	1.9988497	-0.99917	down	x
15.02_612.2206m/z	15.01926667	612.2206115	HMDB0040767	Neoacrimarine E	0.001630891	1.7994195	-0.84753156	down	x
15.03_485.3411m/z	15.02593333	485.3410809	HMDB0118021	CL(8:0/10:0/a-25:0/a-25	0.047622293	1.6458617	0.71884316	up	
15.12_251.1447m/z	15.12211667	251.1446756	HMDB0000145	Estrone	0.004216556	2.4895794	1.315902	up	
15.14_333.2085m/z	15.13926667	333.208538	HMDB0002122	Prostaglandin F3a	0.036308646	1.5187575	-0.6028915	down	
15.16_769.4056m/z	15.15641667	769.4055993	HMDB0265413	PA(20:5(5Z,8Z,11Z,14Z,1	1.79E-04	1.6166866	-0.69304	down	
15.28_773.3081m/z	15.2764	773.308146	HMDB0038230	Bn-NCC-2	0.02339146	1.7252537	0.7868085	up	x
15.29_311.2267m/z	15.29355	311.2266968	CSID7827800	9(S)-HPODE	0.013064166	1.6694373	-0.7393619	down	
15.36_335.2245m/z	15.36211667	335.2244879	HMDB0001483	Prostaglandin F2b	0.004685445	1.7073737	-0.7717789	down	
15.39_474.2086m/z	15.38596667	474.2086253	HMDB0273156	PGP(PGJ2/18:3(9Z,12Z,1	0.026864978	1.522652	0.6065863	up	
15.40_387.1330m/z	15.40311667	387.1329592	HMDB0248126	Aldoxorubicin	0.012375862	1.6478477	0.72058284	up	x
15.40_555.2002m/z	15.3964	555.2002363	HMDB0006825	Tetrahydrofolyl-[Glu](2)	0.04734563	1.948762	-0.9625579	down	
15.41_447.1353m/z	15.41355	447.1353118	CSID390857	Agnuside	0.035010565	1.5735005	-0.65397763	down	x
15.44_579.1145m/z	15.4374	579.1144649	HMDB0302946	Salvianolic acid K	0.04055991	1.6754422	0.74454194	up	x
15.47_265.5763m/z	15.47168333	265.576306	HMDB0259805	Vilanterol	6.23E-04	1.5395107	0.6224719	up	x
15.49_563.1349m/z	15.48883333	563.1349058	HMDB0037408	Neocarlinoside	0.022859981	1.7143718	0.77768004	up	x
15.54_289.9208m/z	15.54026667	289.9208403	HMDB0043599	TG(15:0/22:5(4Z,7Z,10Z,	0.005882935	1.761599	-0.8168855	down	
15.55_493.3555m/z	15.55068333	493.3555073	HMDB0297242	DG(18:3(10,12,15)-OH(9	0.002789387	3.0884347	1.6268758	up	
15.57_335.2258m/z	15.56783333	335.2257787	HMDB0001442	Prostaglandin E1	0.003637792	1.7899964	-0.83995664	down	

15.57_651.2115m/z	15.57455	651.2114899 HMDB0303649	Peonidin 3-rutinoside	0.03722198	3.1198432	-1.6414735 down		x
15.61_390.3349m/z	15.60883333	390.3349393 CSID30776715	11Z-Octadecenylcarnitin	0.00757697	1.5923336	0.6711426 up		
15.68_534.3608m/z	15.67741667	534.3607689 HMDB0011492	LysoPE(0:0/22:2(13Z,16Z	0.02603176	1.771321	0.8248257 up		
15.69_481.2820m/z	15.68783333	481.2820276 CSID35015985	L-Threonyllysyl-L-prolyl-l	0.007242425	1.6133037	-0.690018 down		
15.69_549.2715m/z	15.68783333	549.2714824 CSID30791208	Sarmentoloside	0.003888055	1.5300717	-0.6135993 down		x
15.69_705.3742m/z	15.69456667	705.3742388 HMDB0116518	PGP(a-13:0/i-12:0)	0.039644644	1.5216455	0.6056323 up		
15.69_931.6036n	15.68783333	930.5962916 HMDB0289079	PC(22:6(5Z,8E,10Z,13Z,1	0.037818264	1.5493548	-0.63166755 down		
15.77_407.2817m/z	15.77353333	407.2817015 HMDB0000419	3b,7a,12a-Trihydroxy-5b	0.024920136	1.6973178	0.7632567 up		
15.77_454.2942n	15.77353333	453.2869386 HMDB0296940	DG(20:3(8Z,11Z,14Z)-2O	0.03835908	1.5772458	0.6574075 up		
15.77_535.2428m/z	15.77353333	535.2428145 HMDB0252920	4-(2,2,6,6-Tetramethyl-1	4.64E-04	1.6597059	-0.7309276 down		x
15.77_555.2184m/z	15.77353333	555.2183854 HMDB0034075	Austalide C	0.025966574	2.3830142	1.2527876 up		x
15.77_579.2344m/z	15.77353333	579.2343751 CSID29368373	15-oxo-beta-bilirubin	0.001558833	1.6492906	-0.72184557 down		x
15.79_816.5678n	15.79171667	839.5569962 HMDB0290518	SM(d18:1/6 keto-PGF1al	0.019101772	2.2417703	1.1646384 up		
15.84_391.2869m/z	15.84211667	391.2869357 CSID29131	Ursodeoxycholic acid	0.009280845	1.7121753	-0.7758304 down		
15.84_438.2993n	15.84211667	437.2920351 HMDB0297051	DG(2:0/20:3(6,8,11)-OH(	0.008242647	1.7699916	-0.8237425 down		
15.84_505.2740m/z	15.84211667	505.2740071 HMDB0015593	Ixabepilone	0.026411226	2.1724513	-1.1193238 down		x
15.86_601.1945m/z	15.85925	601.1944676 HMDB0036746	Matsutakeside I	0.003799963	2.7321994	-1.4500628 down		x
15.88_533.2030m/z	15.8764	533.2030038 HMDB0248172	Almorexant	0.002804805	1.6622484	-0.73313594 down		x
15.91_376.2258m/z	15.91068333	376.2257631 HMDB0250613	Cyanoketone	0.001189998	1.5297576	-0.61330307 down		x
15.91_444.2175m/z	15.91068333	444.2174626 HMDB0011471	LysoPE(0:0/14:1(9Z))	9.53E-04	1.5716854	-0.6523125 down		
16.01_299.1872m/z	16.00783333	299.1871811 HMDB0029850	L-Citronellol glucoside	0.001942648	1.6002566	-0.67830324 down		x
16.02_265.1816m/z	16.02496667	265.1815674 HMDB0060055	Tetranor 12-HETE	0.024562092	1.5650793	-0.64623576 down		
16.02_418.2231m/z	16.02496667	418.2231169 HMDB0241393	(2E,4Z)-Tetradeca-2,4-di	0.00759936	1.5094647	-0.59403706 down		
16.02_785.4373m/z	16.02496667	785.4372534 HMDB0264544	PA(19:2(10Z,13Z)/PGJ2)	0.003821015	1.5255303	-0.60931087 down		
16.08_343.1593m/z	16.0764	343.1592506 HMDB0255495	Neamine	0.00757828	1.7102287	-0.77418923 down		x
16.12_615.4570m/z	16.11761667	615.4569704 HMDB0295255	DG(15:0/PGJ2/0:0)	0.03837331	1.5917228	0.67058915 up		
16.16_233.1561m/z	16.16211667	233.1560538 CSID30791620	Kikkanol C	2.75E-04	1.52092	0.6049443 up		x
16.32_579.2293m/z	16.3164	579.2292654 HMDB0005006	Atorvastatin	0.002177383	1.5804448	-0.66033065 down		x
16.33_614.2424n	16.33353333	613.2351221 HMDB0304575	chrysoeriol 7-O-neohesp	0.00924511	1.5265032	-0.6102306 down		x
16.33_644.2241n	16.33353333	665.1987466 CSID24842685	3-[4-Methoxy-3-(3-meth	0.003611861	2.0671008	-1.0476087 down		x
16.33_648.2781m/z	16.33353333	648.2781424 HMDB0260201	(Betas)-beta-(((2S)-2-(4,4	0.011459612	1.7223083	-0.78434336 down		x
16.33_671.2154m/z	16.33353333	671.2153792 CSID391537	Cyclo[D-alpha-aspartyl-L	0.031048154	1.7597153	-0.81534207 down		x
16.34_304.1091m/z	16.34056667	304.1091066 HMDB0304574	hemsleyanoside	0.003897179	1.6457664	-0.71875954 down		x

16.37_640.4155m/z	16.36781667	640.415547	HMDB0035101	Lansioside A	0.026877115	1.6025022	-0.68032634 down	x
16.45_799.4529m/z	16.45353333	799.4529103	HMDB0264999	PA(20:3(5Z,8Z,11Z)/20:4	5.95E-04	1.6428658	-0.7162146 down	
16.46_788.4126n	16.46055	753.3987218	HMDB0041332	Balagyptin	0.01657602	1.796156	0.8449127 up	x
16.47_806.5851m/z	16.47068333	806.5851364	HMDB0290218	CE(LTE4)	0.019195016	2.8465815	-1.5092304 down	
16.49_525.3749m/z	16.48781667	525.374914	CSID115267099	N-(3-Hydroxy-15-methyl	0.001854017	1.8214052	-0.86505187 down	
16.50_1082.7440m/z	16.50496667	1082.743986	CSID113381700	(2S,3R)-2-(Hexacosanoyl	0.03676738	2.276356	-1.1867262 down	x
16.54_738.5293m/z	16.53925	738.5293041	CSID24766663	PC(16:0/P-16:0)	0.004886203	1.5962318	-0.67467016 down	
16.54_792.4900m/z	16.53925	792.4899967	HMDB0261443	PE(20:4(7E,9E,11Z,13E)-3	0.007658529	1.9276978	-0.94687885 down	
16.56_1047.5897m/z	16.5564	1047.589657	HMDB0274773	PGP(a-25:0/PGE1)	0.014281583	1.7224013	-0.78442127 down	
16.56_519.2353m/z	16.5564	519.2353129	HMDB0039001	Purothionin AII	0.044313807	1.8219109	-0.86545235 down	x
16.56_806.5120m/z	16.5564	806.5119881	HMDB0113476	PE-NMe(20:4(8Z,11Z,14Z	3.68E-04	1.7393585	-0.7985553 down	
16.56_874.5028m/z	16.5564	874.5028225	HMDB0284321	PE(22:5(4Z,7Z,10Z,13Z,1	0.008360573	2.1651566	-1.1144713 down	
16.56_934.5084m/z	16.5564	934.5084406	CSID30786288	CDP-dipalmitoyl-sn-glyce	0.008269195	1.9016781	-0.92727304 down	x
16.56_979.6014m/z	16.5564	979.6014104	HMDB0276024	PGP(i-24:0/20:3(6,8,11)-	0.020224841	1.5144794	-0.59882194 down	
16.61_820.5420m/z	16.60781667	820.5420392	HMDB0289657	PC(P-18:1(11Z)/PGE2)	0.021059707	1.6455169	-0.7185408 down	
16.64_249.1539m/z	16.6421	249.1539485	HMDB0243865	1-Dodecanesulfonic acid	0.010280917	1.9302367	0.94877774 up	x
16.66_378.2430m/z	16.65925	378.2430406	HMDB0000277	Sphingosine 1-phosphate	0.001189006	1.549945	-0.632217 down	
16.66_593.1954m/z	16.66068333	593.1954013	HMDB0252147	Exherin	0.042970274	1.7576227	-0.8136254 down	x
16.70_673.5233m/z	16.69503333	673.523294	HMDB0007469	DG(20:3(5Z,8Z,11Z)/22:6	0.034610577	1.6369916	0.71104693 up	
16.70_693.2770m/z	16.69503333	693.276955	HMDB0303308	Starch, pregelatinized	0.034590486	1.8112311	0.85697067 up	x
16.71_747.5481m/z	16.71218333	747.5480634	HMDB0296455	DG(21:0/20:4(5Z,8Z,11Z,	0.033190206	1.5898931	0.66892976 up	
16.74_289.1851m/z	16.74496667	289.1850818	CSID4511170	octinoxate	0.005953359	1.5225928	-0.60653013 down	x
16.80_386.1266m/z	16.79638333	386.1266074	HMDB0031401	Dihydroxycitracridone I	2.00E-04	1.8166599	0.86128837 up	x
16.80_736.5182m/z	16.79638333	736.51819	HMDB0007898	PC(14:0/P-18:1(9Z))	0.029406292	1.6123942	-0.6892045 down	
16.82_728.4497m/z	16.81503333	728.449699	HMDB0280887	PS(14:0/18:3(10,12,15)-(	0.04082652	1.5773349	0.657489 up	
16.85_377.1641m/z	16.84781667	377.1640728	HMDB0252320	Fluanisone	0.011275088	1.5844331	-0.6639667 down	x
16.85_874.5622m/z	16.84781667	874.5622038	HMDB0283991	PE(22:6(5Z,8E,10Z,13Z,1	0.006024883	1.9570612	-0.96868885 down	
16.88_543.2251m/z	16.88361667	543.2251232	HMDB0041308	Italidipyrone	0.00361613	2.1735206	1.1200337 up	x
16.95_986.5561m/z	16.95066667	986.5560643	HMDB0260048	Zotarolimus	0.03772573	1.7011077	-0.7664745 down	x
16.97_286.2368m/z	16.9694	286.2368218	HMDB0061656	3-Hydroxytetradecanoic	0.045250595	1.6048292	-0.6824197 down	
16.97_320.2380n	16.96781667	319.2307594	HMDB0011134	5-HETE	0.00473435	1.5408114	-0.6236903 down	
16.97_768.5570m/z	16.96781667	768.5570377	HMDB0261875	PE(18:1(12Z)-O(9S,10R)/	0.010740837	2.3707936	1.24537 up	
17.05_239.1687m/z	17.04781667	239.1686859	CSID12630	Tetradecanedioic acid	4.41E-04	1.6811986	-0.74949014 down	

17.06_758.5026m/z	17.06496667	758.5026008	HMDB0260821	PE(20:3(8Z,11Z,14Z)-2OH	1.11E-04	1.9888633	-0.99194413	down	
17.07_413.2657m/z	17.07225	413.2657432	HMDB0241230	Dodec-6-enedioylcarnitii	0.02814506	1.5780616	0.6581535	up	
17.09_113.1327m/z	17.0894	113.1327103	HMDB0001183	Octanol	0.044138268	1.7475712	0.80535126	up	x
17.10_984.6172m/z	17.09925	984.6172024	HMDB0249224	Biolimus A9	0.04545859	1.6472609	-0.72006905	down	x
17.17_1030.5325m/z	17.16781667	1030.532463	HMDB0032002	Dehydrotomatine	0.013111861	1.751642	0.80870795	up	x
17.17_535.2956m/z	17.16781667	535.2956317	HMDB0242023	N-Docosahexaenoyl Tryp	3.75E-04	1.7262905	0.7876752	up	
17.17_894.5496m/z	17.16781667	894.5496168	HMDB0286454	PC(TXB2/18:1(9Z))	0.00778772	1.796045	0.8448234	up	
17.18_831.0638m/z	17.1752	831.0637704	HMDB0039263	Tercatain	0.031772695	1.6574695	0.7289823	up	x
17.20_1054.6311m/z	17.2021	1054.631136	CSID390114	Lubomycine B	0.018035218	1.5377666	0.6208365	up	x
17.20_824.5484m/z	17.2021	824.5484237	HMDB0285371	PE(PGF1alpha/P-18:0)	0.020009123	2.0650053	1.0461454	up	
17.22_1030.6301m/z	17.21925	1030.630098	HMDB0270979	PG(a-25:0/LTE4)	0.001703991	1.7671009	0.82138443	up	
17.29_303.2368m/z	17.28781667	303.2367903	CSID392692	Arachidonic acid	5.59E-04	1.9443179	0.95926416	up	
17.29_345.2479m/z	17.28781667	345.2478507	CSID4436580	16,16-Dimethylprostagla	0.003810106	1.546448	0.6289583	up	
17.29_466.3186n	17.28781667	487.2932264	HMDB0241986	N-Linoleoyl Tryptophan	2.06E-04	1.7445095	0.8028214	up	
17.29_750.5494m/z	17.28781667	750.5494267	HMDB0009445	PE(20:4(8Z,11Z,14Z,17Z)	0.016415423	2.0176635	-1.0126855	down	
17.29_776.5557m/z	17.28781667	776.555713	HMDB0009266	PE(20:1(11Z)/20:3(8Z,11	0.005903326	1.8483397	0.8862299	up	
17.32_873.5742m/z	17.3221	873.5741763	CSID113377442	(2R)-3-({[(2S)-2,3-Dihydr	0.02766816	1.6687335	-0.73875356	down	x
17.34_279.2367m/z	17.33925	279.2367215	CSID558800	Ricinoleic Acid	5.91E-04	1.7730231	0.82621133	up	x
17.36_550.3076m/z	17.35638333	550.3075657	CSID113376756	O-(Hydroxy{(2R)-2-hydrc	1.36E-04	1.8222393	0.8657124	up	x
17.39_600.3238m/z	17.39066667	600.3238027	HMDB0288844	PC(2:0/20:3(8Z,11Z,14Z)	0.01354588	1.6680671	-0.7381773	down	
17.39_816.0630n	17.39246667	781.0491832	HMDB0303059	Brevilagin I	0.037267357	1.7230186	0.7849383	up	x
17.41_495.7495m/z	17.40781667	495.7494525	HMDB0290819	CDP-DG(16:0/18:1(12Z)-	0.020129798	2.1426706	-1.09941	down	
17.43_630.7098m/z	17.42675	630.7097677	HMDB0250920	Dechlorane plus	0.045600045	1.5193493	0.60345364	up	x
17.44_1198.7685m/z	17.44388333	1198.768487	HMDB0242417	Deoxycholyltryptophan	0.043987982	1.5747417	0.65511525	up	
17.46_413.3273m/z	17.45925	413.3272633	HMDB0092907	DG(8:0/13:0/0:0)	0.03573414	1.6740295	-0.74332494	down	
17.51_1104.7524m/z	17.51066667	1104.752397	CSID113381716	(2S,3R)-3-Hydroxy-2-(tet	0.03784041	1.7270608	0.7883189	up	x
17.55_603.8950m/z	17.54686667	603.8949998	HMDB0249816	Ceramide trihexosides	0.009006283	1.5548614	0.636786	up	x
17.55_711.5276m/z	17.54686667	711.5275909	HMDB0115070	PA(20:0/18:2(9Z,12Z))	0.04101323	1.6366522	0.7107478	up	
17.63_534.3195m/z	17.63066667	534.3195494	HMDB0252830	Gly-Pro-Arg-Pro-Lys	7.10E-05	2.0940244	1.0662782	up	
17.65_237.0831m/z	17.64973333	237.083071	CSID4472029	5,11-Dodecadiynoic acid	0.048316725	1.6462815	-0.71921104	down	x
17.73_826.5609m/z	17.73351667	826.560904	HMDB0261913	PE(PGD1/20:0)	0.009569609	2.066423	1.0471356	up	
17.75_766.5424m/z	17.75066667	766.5423539	HMDB0286156	PC(18:2(9Z,11E)+=O(13),	0.019493807	1.5761011	0.65636003	up	
17.89_206.1254m/z	17.88985	206.1254461	CSID57505950	1-Hydroxy-3-(phosphonc	0.048966352	1.5388634	0.6218652	up	x

17.92_675.5378m/z	17.92413333	675.5378422 HMDB0290165	Cer(t18:0/PGJ2)	0.013840365	1.7963321	0.8450541 up		
18.00_443.1999m/z	18.0021	443.1999269 CSID4574282	6-dehydrotestosterone 1	0.022189088	1.6449612	-0.7180536 down		
18.04_188.5876m/z	18.03853333	188.5876005 HMDB0241053	3-methyloctanedioylcarr	0.015383177	1.5125028	0.5969378 up		
18.10_1027.7711m/z	18.10495	1027.771097 HMDB0053548	TG(20:2n6/22:4(7Z,10Z,1	0.015598347	1.5556755	-0.6375412 down		
18.11_266.0782m/z	18.10711667	266.0781795 HMDB0029494	Formononetin 7-(6''-met	0.033717543	1.5710018	0.6516848 up		x
18.11_447.1974m/z	18.10711667	447.1974203 HMDB0034159	Acetyl tributyl citrate	0.032331813	1.5507339	0.63295114 up		x
18.19_597.4167m/z	18.19066667	597.416722 HMDB0294624	DG(20:4(5Z,8Z,11Z,14Z)-	0.042454578	1.637902	0.71184903 up		
18.21_311.3034m/z	18.21006667	311.3033774 HMDB0040940	Capsiamide	0.02565091	2.3241692	-1.2167151 down		x
18.24_377.2236m/z	18.24435	377.2235583 HMDB0114829	PA(15:0/22:5(7Z,10Z,13Z	0.019889608	1.5202392	0.60429835 up		
18.24_406.1684n	18.24435	407.1756597 HMDB0249317	Bms-378806	0.00145388	1.577001	0.6571835 up		x
18.24_623.2196m/z	18.24435	623.2195627 HMDB0255552	Netupitant	0.004590423	2.415301	1.272203 up		x
18.26_1091.7214m/z	18.25923333	1091.721416 HMDB0004891	Ganglioside GA2 (d18:1/	0.04327728	1.515624	-0.5999119 down		
18.26_1159.7089m/z	18.25923333	1159.708851 HMDB0011929	Ganglioside GM3 (d18:1,	0.043203637	1.5030584	-0.5879011 down		
18.29_575.1872m/z	18.29351667	575.1871701 HMDB0301813	Lappaol C	0.04725245	1.567477	0.64844424 up		x
18.29_697.4493m/z	18.29351667	697.4492862 HMDB0263165	PA(20:3(8Z,11Z,14Z)-O(5	0.014692624	1.6492959	0.7218503 up		
18.29_759.4169m/z	18.29351667	759.416917 HMDB0116572	PGP(i-14:0/i-15:0)	0.04513775	1.5696528	0.65044546 up		
18.31_473.1529m/z	18.31293333	473.1529124 HMDB0301002	3-OxoUndecanoyl-CoA	0.026679723	1.6492418	0.72180295 up		
18.31_501.1221m/z	18.31293333	501.1220798 HMDB0302318	Lappaphen A	0.034155052	1.9465295	0.9609042 up		x
18.36_457.2675m/z	18.36435	457.2674806 HMDB0272543	PGP(20:3(8Z,11Z,14Z)-2(	0.00430206	1.5979414	-0.6762145 down		
18.48_745.2986m/z	18.48445	745.2985835 HMDB0039694	Physagulin G	0.044200428	1.5279815	-0.6116271 down		x
18.48_812.2890m/z	18.48445	812.2890409 HMDB0015657	Nilvadipine	0.003693058	1.5283422	-0.6119676 down		x
18.50_549.6684m/z	18.5016	549.6683819 HMDB0011656	2,4,7,10,13,16,19-Docos	0.01296794	1.7493405	-0.80681115 down		
18.55_269.1111m/z	18.55303333	269.1111221 HMDB0266541	PA(2:0/18:1(12Z)-O(9S,1	0.04887398	1.7551763	0.81161594 up		
18.69_757.5339n	18.6878	738.5161069 HMDB0261253	PE(18:1(9Z)-O(12,13)/18	0.017006744	1.5787288	0.65876335 up		
18.72_849.5734n	18.72208333	870.5480996 HMDB0282100	PS(18:1(12Z)-2OH(9,10)/	0.009487489	1.8545364	0.89105856 up		
18.79_1001.5157n	18.79065	1000.508387 HMDB0115969	CDP-DG(18:2(9Z,12Z)/18	0.024781633	1.8191069	0.86323035 up		
18.81_804.5744m/z	18.8078	804.5744067 HMDB0112709	PS(22:0/15:0)	0.015871027	1.6054237	0.6829541 up		
18.82_1025.5438n	18.82493333	1006.525974 HMDB0293001	CDP-DG(a-17:0/18:1(12Z	0.021550685	1.5483145	0.6306985 up		
18.82_1068.4946m/z	18.82493333	1068.494627 HMDB0292983	CDP-DG(a-17:0/20:4(6Z,8	0.02673066	2.056641	1.04029 up		
18.82_1074.5133m/z	18.82493333	1074.513285 HMDB0291418	CDP-DG(PGF2alpha/18:2	0.04091238	1.6821237	0.7502838 up		
18.84_715.3782m/z	18.84208333	715.3782269 CSID4444702	Spinoside A	0.001703549	1.5388222	-0.6218265 down		x
18.84_959.6031m/z	18.83898333	959.6031071 HMDB0284926	PE(DiMe(13,5)/22:6(5Z,8	0.003765622	1.6612451	-0.73226494 down		
18.84_986.5969m/z	18.83898333	986.5969217 HMDB0276996	PI(PGJ2/20:0)	0.003069517	1.5890013	-0.66812027 down		

18.91_369.1643m/z	18.90755	369.1642818 HMDB0249108	beta-Casomorphin-7	0.019789355	2.1476078	1.1027305 up	x
18.94_1156.3375m/z	18.94183333	1156.337519 HMDB0041158	Cyanidin 3-(6-feruloyl-2'	0.020794889	2.2885644	-1.1944429 down	x
18.99_395.1099m/z	18.99065	395.1098658 HMDB0040912	N-[(5-Hydroxy-2-pyridiny	0.002595353	2.0397203	-1.0283713 down	x
19.01_265.1490m/z	19.0078	265.1490466 CSID8448	laurilsulfate	0.02554543	1.5533458	-0.635379 down	x
19.01_333.1386m/z	19.0078	333.13863 HMDB0041826	Amifloxacin	0.021937937	1.6039795	-0.68165565 down	x
19.21_401.1585m/z	19.20896667	401.1584738 HMDB0035166	Cinncassiol C	0.010642362	1.8225914	-0.8659912 down	x
19.26_463.7392m/z	19.26308333	463.7391573 HMDB0282764	PS(PGD1/20:4(8Z,11Z,14	0.032147795	1.5534552	-0.63548064 down	
19.26_471.1045m/z	19.26308333	471.104485 HMDB0260340	Cytidine-5'-diphosphoch	0.049987555	1.6191673	-0.69525206 down	x
19.28_307.1626m/z	19.27753333	307.1625846 CSID5898	Androstenedione	0.009189721	2.1289303	-1.0901288 down	
19.31_1007.5005n	19.31451667	972.486614 HMDB0293508	CDP-DG(i-14:0/20:3(8Z,1	0.007183861	1.9877077	-0.9911056 down	
19.35_1000.5101m/z	19.3488	1000.510065 HMDB0291229	CDP-DG(18:1(9Z)/18:2(1	0.026381325	1.7028009	-0.7679097 down	
19.35_1163.6837m/z	19.3488	1163.683705 HMDB0252932	Gramicidin S	0.02374098	2.1201072	-1.0841372 down	x
19.43_206.1648n	19.43461667	207.1720384 HMDB0013816	2,4-Di-tert-butylphenol	0.045581844	1.6301081	0.7049676 up	x
19.45_218.5758m/z	19.45176667	218.5758404 HMDB0012575	13E-Tetranor-16-carboxy	0.023378428	2.1023138	1.071978 up	
19.45_275.0792m/z	19.45176667	275.0791886 HMDB0039930	(S)-Multifidol 2-[apiosyl-	0.012072762	1.5951664	0.67370695 up	x
19.45_328.2247m/z	19.45176667	328.2246588 HMDB0032941	Momorcharaside B	0.031007402	1.7070283	0.77148694 up	x
19.45_486.1507m/z	19.45176667	486.1507454 HMDB0012607	18-Carboxy-dinor-LTE4	0.04960649	1.9841218	0.9885006 up	
19.47_336.2187m/z	19.4689	336.2187331 HMDB0262853	PA(20:3(8Z,11Z,14Z)-O(5	0.031121302	1.8469414	0.88513803 up	
19.47_393.2178m/z	19.4689	393.2178016 CSID4446239	19(R)-hydroxy Prostaglar	0.017506853	1.8912548	0.9193437 up	
19.47_783.0629m/z	19.4689	783.062886 HMDB0030437	Emblicanin A	0.007820418	2.0882106	-1.0622672 down	x
19.50_328.3184m/z	19.50318333	328.318357 HMDB0062673	2-(4-methyl-1-piperaziny	0.007052444	3.0262184	1.5975161 up	
19.70_1162.3732m/z	19.7033	1162.373185 HMDB0041489	5''-(4-Hydroxy-(E)-cinnar	0.014148318	1.887739	-0.91665924 down	x
19.70_705.9932m/z	19.7033	705.9932371 HMDB0256336	Perfluorotridecanoic acid	0.04838672	1.9394811	0.9556707 up	x
19.72_813.6730m/z	19.72043333	813.6730068 HMDB0042329	TG(14:0/16:1(9Z)/22:6(4	0.030120747	1.5237108	0.6075891 up	
19.72_949.6051m/z	19.72043333	949.6051298 HMDB0284580	PE(24:1(15Z)/LTE4)	0.001119191	1.6130213	-0.68976545 down	
19.81_465.3121m/z	19.80895	465.3121398 HMDB0241986	N-Linoleoyl Tryptophan	0.03445318	1.6291856	0.7041509 up	
19.83_762.5156m/z	19.8261	762.51558 HMDB0261501	PE(20:3(6,8,11)-OH(5)/1	0.022530861	1.6376882	0.7116607 up	
19.99_410.7577m/z	19.99483333	410.7577083 HMDB0285824	PC(15:0/PGJ2)	0.00598081	1.633424	0.70789933 up	
2.44_252.0909m/z	2.442383333	252.0908933 HMDB0246178	4-Aminophenylmannosid	0.043501526	1.5978954	-0.676173 down	x
2.44_342.1220m/z	2.442383333	342.1219826 HMDB0041208	De-O-methylsimmondsir	0.013523366	1.8359135	-0.8764981 down	x
2.44_402.0785m/z	2.442383333	402.078456 HMDB0006806	Propinol adenylate	0.002315852	1.9654474	-0.97485775 down	x
2.46_400.0822m/z	2.459516667	400.0822031 HMDB0001066	S-Lactoylglutathione	0.028707381	1.6492099	-0.721775 down	
2.46_410.1100m/z	2.459516667	410.1099832 HMDB0014958	Tadalafil	0.007604873	1.8654987	-0.8995613 down	x

2.63_270.0987m/z	2.63095	270.0986958 HMDB0243858	1-Deoxy-1-morpholino-D	0.00800997	1.7436893	-0.802143 down	x
2.63_376.1018m/z	2.63095	376.1017526 HMDB0256631	Plafibride	0.010254408	1.7389202	-0.7981917 down	x
2.63_428.1186m/z	2.63095	428.1186225 CSID35014630	gamma-Glutamyl-gamm	0.004177824	1.7101542	-0.7741264 down	
2.63_659.1298m/z	2.63095	659.1298362 CSID24845678	3,5,8-Trihydroxy-2-(4-hy	0.006442026	1.7452257	-0.80341357 down	x
2.65_247.0439m/z	2.648083333	247.0439048 HMDB0240292	2,3,4,5,6,7-Hexahydroxy	4.88E-05	2.9993691	-1.5846591 down	
2.68_130.0857m/z	2.682366667	130.0857363 CSID834	Leucine	0.004606862	1.628603	-0.7036349 down	
2.68_293.1487n	2.682366667	292.1414609 HMDB0241652	3,4,5-Trihydroxypentanc	0.007287748	1.7056208	-0.7702969 down	
2.68_329.1267n	2.682366667	350.1013642 HMDB0032805	N-trans-Feruloyloctopan	0.00394865	1.6662	-0.7365616 down	x
2.68_331.1252n	2.682366667	352.0998381 HMDB0246884	5'-O-beta-D-Glucosylpyri	0.004561484	1.7390279	-0.798281 down	x
2.68_360.1298m/z	2.682366667	360.1297691 HMDB0041208	De-O-methylsimmondsir	0.006657583	1.6585094	-0.7298871 down	x
2.68_422.1020m/z	2.682366667	422.1020232 HMDB0257230	Rilmakalim	0.010225421	1.7039802	-0.76890856 down	x
2.68_480.1438m/z	2.682366667	480.1437731 HMDB0015349	Halofantrine	0.038290817	1.6473699	-0.7201645 down	x
2.70_202.1093m/z	2.699516667	202.1092883 HMDB0240626	1-Carboxyethylleucine	0.006761856	1.7270211	-0.7882857 down	x
2.70_244.1219m/z	2.699516667	244.1219497 HMDB0241651	3-Oxobutanoylcarnitine	0.004386674	1.7710193	-0.82457995 down	
2.70_286.0642m/z	2.699516667	286.0642243 HMDB0014572	Streptozocin	0.04420568	1.6932672	-0.7598097 down	x
2.70_418.1715m/z	2.699516667	418.1715463 CSID389923	Meropenem	0.01658567	1.6664926	-0.73681486 down	x
2.70_486.1604m/z	2.699516667	486.1603969 HMDB0003324	Biotripyrrin-b	0.006709321	1.7675397	-0.82174265 down	
2.70_586.2966n	2.699516667	585.2892907 HMDB0251322	Dihydroouabain	0.00471891	2.2505817	-1.170298 down	x
2.70_665.2317m/z	2.699516667	665.23173 HMDB0253979	Lapaquistat acetate	0.007987618	1.948897	-0.9626578 down	x
2.72_476.1332m/z	2.71665	476.1332448 HMDB0303639	Malvidin 3-rhamnoside	0.001184881	1.8459605	-0.8843717 down	x
2.80_380.0988m/z	2.802366667	380.0988481 HMDB0010336	Epinephrine glucuronide	1.09E-04	2.6057134	-1.3816783 down	
2.85_228.0346m/z	2.8538	228.03458 HMDB0252436	Forchlorfenuron	0.00175886	2.5443811	-1.3473148 down	x
2.85_249.0539m/z	2.848316667	249.0538627 HMDB0039166	Cajaquinone	0.004181213	1.6978347	-0.763696 down	x
3.22_315.1917m/z	3.21975	315.1917087 HMDB0241670	(4E)-3-Hydroxyhex-4-enc	0.008641013	1.946545	-0.9609157 down	
3.40_513.1884m/z	3.39665	513.1884142 HMDB0032761	Nb-trans-Feruloylserotor	0.007258975	1.7201211	-0.78251016 down	x
3.43_236.0951m/z	3.430933333	236.0950907 HMDB0062175	N-Lactoylphenylalanine	0.007019697	1.6513504	-0.7236462 down	
3.43_327.1333n	3.430933333	326.1260582 HMDB0037846	N-(1-Deoxy-1-fructosyl)p	0.006601415	1.6744571	-0.74369335 down	x
3.43_363.1120n	3.430933333	384.0866913 CSID10512124	7,8-didemethyl-8-hydrox	0.004217519	1.7723732	-0.8256824 down	x
3.43_394.1145m/z	3.430933333	394.1145317 HMDB0014577	Prochlorperazine	0.004039166	1.6914251	-0.75823927 down	x
3.43_410.0857m/z	3.430933333	410.085659 HMDB0304508	trans-zeatin riboside mo	0.006721542	1.739947	-0.7990433 down	x
3.43_438.1420m/z	3.430933333	438.1420376 HMDB0035030	Amygdalin	0.031256817	1.6543909	-0.7263002 down	x
3.43_462.1029m/z	3.430933333	462.1028877 CSID23107017	1D-myo-inositol 2-acetai	0.001797359	1.8666455	-0.9004479 down	x
3.43_654.2671n	3.430933333	653.2597918 HMDB0000644	Coproporphyrin IV	0.026302328	1.7826931	-0.8340584 down	x

3.45_217.0306m/z	3.448066667	217.0305718	CSID26333255	L-Galactonic acid	2.30E-04	2.4804153	1.3105817 up	x
3.45_239.0135m/z	3.448066667	239.0134942	CSID575	cystine	1.42E-04	2.7568254	1.4630079 up	
3.52_323.0415m/z	3.516633333	323.0414887	HMDB0060017	Pyrogallol-2-O-glucuroni	0.040874436	1.7289021	0.7898562 up	
3.67_256.1292m/z	3.66545	256.1292408	HMDB0034684	Thalictroidine	0.04005144	1.5833503	-0.66298044 down	x
4.00_218.0383n	3.996616667	217.0310221	HMDB0303730	Sodium gluconate	5.97E-04	2.4053323	1.2662362 up	x
4.09_323.0414m/z	4.093766667	323.0413751	HMDB0060017	Pyrogallol-2-O-glucuroni	0.003501365	2.2296894	1.1568427 up	
4.14_438.1977m/z	4.139733333	438.1976898	HMDB0039953	1-(3-Methylbutanoyl)-6-	0.004976601	1.5982383	-0.67648256 down	x
4.16_456.2083m/z	4.156866667	456.2083045	HMDB0003752	LysoPC(10:0/0:0)	0.001171011	1.7388389	-0.79812425 down	
4.19_114.0667m/z	4.19115	114.0667057	HMDB0000064	Creatine	0.046039097	1.5991646	-0.67731845 down	
4.40_173.0382m/z	4.396866667	173.0381964	HMDB0029036	Serylcysteine	0.041621197	1.628064	0.7031574 up	x
4.41_256.9934m/z	4.414	256.9934028	HMDB0240569	Urolithin-3-sulfate	0.014640258	2.068999	1.048933 up	x
4.45_149.5124m/z	4.448283333	149.5124338	HMDB0257542	(5R)-3-Ethylsulfanyl-6-(1	0.007363007	1.7925082	0.84197974 up	x
4.63_496.1045n	4.625183333	495.0972656	HMDB0250829	Daf-FM DA	0.048860814	1.58978	-0.6688271 down	x
4.66_573.1726m/z	4.659466667	573.1725762	HMDB0302298	Arctignan B	0.007890629	1.8692504	-0.90245986 down	x
4.69_257.1034m/z	4.69375	257.1033702	HMDB0034263	Triethyl citrate	0.010381728	1.6227186	-0.69841284 down	x
4.71_324.0765m/z	4.710883333	324.0764799	HMDB0240379	HHPAA glucuronide	0.009905502	1.5028952	-0.5877444 down	x
4.75_173.0404m/z	4.745183333	173.0404042	CSID10254628	Ribitol	4.62E-04	2.4754574	1.3076952 up	x
4.75_399.1153m/z	4.745183333	399.1152832	CSID133675	Isouvaretin	0.007151401	2.0024865	1.0017924 up	x
4.76_241.0315m/z	4.762316667	241.031549	HMDB0059775	3-Hydroxy-3-carboxymet	0.010624834	2.0082293	1.005924 up	
4.81_406.1354m/z	4.81375	406.1354156	HMDB0254740	Minoxidil Glucuronide	0.038738627	1.6149528	-0.691492 down	x
4.83_179.1888m/z	4.830883333	179.1888209	CSID7877401	Butanal, dibutylhydrazor	0.037107203	1.6584555	-0.7298403 down	x
4.83_240.0904m/z	4.830883333	240.0904384	HMDB0013134	Fumarycarnitine	0.033202693	1.837562	-0.8777929 down	
4.83_449.0956m/z	4.830883333	449.0956129	CSID119550	Gyrophoric acid	8.54E-04	1.7859513	-0.8366927 down	x
5.23_375.1874m/z	5.231133333	375.18742	HMDB0241866	(8S)-8-Amino-7-oxonona	0.025109237	1.5224904	-0.6064331 down	
5.31_421.1861m/z	5.305166667	421.1861156	CSID83309	ISOVALTRATE	0.003942566	1.6059674	-0.6834426 down	x
5.31_467.1103m/z	5.305166667	467.1103376	CSID23316458	trichodermamide C	1.56E-05	2.0628235	-1.0446204 down	x
5.44_446.9971m/z	5.4423	446.9970553	CSID24845593	2-(3,4-Dihydroxyphenyl)	0.03377818	1.7639273	-0.81879115 down	x
5.77_178.0008m/z	5.774	178.0008153	HMDB0250115	Chlorofluoromethane	0.004368935	1.6939617	0.7604013 up	x
5.77_233.0966m/z	5.774	233.0966139	HMDB0000670	Homo-L-arginine	5.23E-06	2.0149727	-1.0107603 down	
5.80_437.0908m/z	5.8023	437.0907654	HMDB0001520	Flavin mononucleotide	5.67E-04	1.9248943	-0.9447792 down	
6.02_256.0679m/z	6.019433333	256.0678874	CSID5284	tipepidine	0.016914412	2.2327628	-1.1588299 down	x
6.09_290.0678m/z	6.088	290.0677578	HMDB0010319	Indoxyl glucuronide	0.006190945	1.5719658	-0.65256983 down	
6.68_348.1897m/z	6.67685	348.1897167	HMDB0275649	PGP(LTE4/i-20:0)	0.021746377	2.525283	-1.3364451 down	

7.09_230.9985m/z	7.0937	230.9984683	HMDB0132500	2-[4-(sulfooxy)phenyl]ac	0.03976843	1.5663294	0.6473876	up	x
8.07_498.1574n	8.0651	497.1501556	HMDB0035664	Dukunolide B	0.003774122	1.546287	-0.6288081	down	x
9.07_237.0782m/z	9.0708	237.0782387	HMDB0254201	Lumiflavin	0.002910139	2.4322584	1.2822964	up	x
9.40_358.0541m/z	9.3965	358.0541199	HMDB0013189	3-Indole carboxylic acid [illegible]	0.004395961	1.881724	-0.912055	down	
9.65_315.1030m/z	9.653633333	315.1030338	CSID22370267	Karanjachromene	0.017834058	1.5427657	0.625519	up	x
9.80_197.0434m/z	9.802533333	197.0433874	HMDB0041749	Isoferuloyl C1-glucuronic	0.038995784	1.578845	-0.65886956	down	x

Supplementary Table 3: Endogenous metabolites. Paired t.test, no correction p-value ≤ 0.05, FC 1.5.

Compound	Retention time (min)	Mass	Accepted Compound ID	Accepted Description	p ([Post] Vs [Pre])	FC (abs) ([Post] Vs [Pre])	Log FC ([Post] Vs [Pre])	Regulation ([Post] Vs [Pre])
0.79_877.9494m/z	0.790983333	877.9493894	HMDB0248429	Angiotensinogen	0.044428974	1.6447862	0.7179001	up
0.83_854.4030m/z	0.825266667	854.4029707	HMDB0116068	CDP-DG(a-13:0/i-12:0)	0.023055285	1.7101332	0.7741087	up
0.88_846.1632m/z	0.8767	846.1631912	HMDB0300835	4-Methylpentanoyl-CoA	0.020493709	1.7327727	-0.7930824	down
0.88_964.7675m/z	0.8767	964.7675096	HMDB0114629	PE-NMe2(24:0/24:0)	0.037907135	1.5166286	-0.60086787	down
0.89_250.0969m/z	0.893833333	250.0969237	HMDB0001983	5'-Deoxyadenosine	0.015466255	1.7051629	-0.76990956	down
0.89_257.0583m/z	0.893833333	257.0582979	HMDB0029045	Serylmethionine	0.014568478	1.6206636	-0.6965847	down
0.89_388.9446m/z	0.893833333	388.9445874	HMDB0062643	5-O-phosphonato-alpha-	0.012042287	2.1803746	-1.124576	down
0.91_160.0969m/z	0.905366667	160.0968871	HMDB0011757	N-Acetylvaline	0.0245198	1.6515797	0.7238466	up
0.95_267.0751m/z	0.945266667	267.0751432	HMDB0013713	N-Acetyltryptophan	0.011192644	1.7230735	-0.78498423	down
1.00_111.0066m/z	0.996683333	111.0065703	HMDB0001051	Glyceraldehyde	0.03169592	2.3296943	1.2201406	up
1.33_116.0705m/z	1.333833333	116.0705203	HMDB0000883	L-Valine	0.013315325	1.8026835	-0.8501461	down
1.33_188.0946m/z	1.333833333	188.0945874	HMDB0062181	N-Lactoylvaline	0.004938704	1.8232882	-0.86654264	down
1.33_307.0377m/z	1.333833333	307.0377421	CSID58574	Deoxyuridine monophos	0.028361771	1.6610477	-0.7320935	down
1.33_508.0585m/z	1.333833333	508.0585216	HMDB0006744	3-Carboxy-1-hydroxypro	0.011763437	1.9478915	-0.9619133	down
1.42_361.1960m/z	1.41975	361.1959818	HMDB0003933	Pentosidine	0.04367991	1.7133973	-0.7768597	down
1.44_290.0912m/z	1.436683333	290.0911958	HMDB0000230	N-Acetylneuraminic acid	5.06E-04	1.9973924	-0.9981178	down
1.57_128.0337m/z	1.573833333	128.0336871	HMDB0000148	L-Glutamic acid	0.02684131	1.7142011	-0.77753633	down
1.57_342.0435m/z	1.573833333	342.0435302	CSID19992713	N-[3-Carboxy-2-(carboxy	0.020188767	2.2722666	-1.1841321	down
1.64_196.0249m/z	1.6424	196.024876	CSID88007	N-Acetylaspartic acid	0.023533659	1.5408987	-0.62377197	down
1.73_342.0446m/z	1.7281	342.0446206	HMDB0013220	Beta-Citryl-L-glutamic aci	0.009920922	2.0599792	-1.0426297	down
10.69_503.2624m/z	10.68825	503.2624468	HMDB0011875	Ganglioside GM1 (d18:0/	0.044839337	1.5775892	-0.6577215	down
11.10_414.3364m/z	11.09966667	414.3363998	HMDB0062342	N-Stearoyl phenylalanine	0.014545579	2.0332472	-1.0237856	down
12.43_209.0878m/z	12.43073333	209.0877867	CSID120970	Alanylvaline	0.022987157	1.7256304	0.7871235	up
13.37_304.0391m/z	13.36786667	304.0391131	CSID5901	cytidine 5′-monophosph	0.029593918	1.7317793	-0.7922551	down
13.39_429.2015m/z	13.385	429.2015059	HMDB0114745	LysoPA(18:4/0:0)	0.005314179	1.5125154	-0.5969499	down
13.48_394.2754m/z	13.4827	394.2753602	HMDB0242022	N-Docosahexaenoyl Thre	0.046887442	1.6711013	0.7407992	up
13.79_309.1311m/z	13.7856	309.1311062	HMDB0000050	Adenosine	0.015093282	1.5221747	0.60613394	up
14.34_321.1498m/z	14.33928333	321.1498352	HMDB0000010	2-Methoxyestrone	8.37E-05	1.5384978	-0.62152237	down
14.37_460.2948m/z	14.37356667	460.294833	CSID24607453	7-{(3S,4S,5R)-4-[(1E)-3-H	0.004327671	1.8566513	-0.8927029	down
14.45_386.3094m/z	14.44855	386.3093582	HMDB0062339	N-Palmitoyl phenylalanin	0.045934897	1.8626549	-0.8973604	down
14.53_435.2732m/z	14.52785	435.2731749	HMDB0296902	DG(20:4-OH(9)/0:0/2:0)	5.66E-04	1.6587929	-0.7301337	down
14.56_253.1476m/z	14.56213333	253.1475528	HMDB0241923	N-Palmitoyl Cysteine	5.67E-04	1.512098	-0.5965516	down
14.66_321.1496m/z	14.66498333	321.1495623	HMDB0006772	Adrenosterone	5.72E-06	1.6040126	-0.68168545	down
14.84_295.1571m/z	14.83641667	295.1571113	CSID13628077	3-Hydroxytetradecanedi	9.76E-05	1.6547801	-0.72663957	down
14.91_170.0956m/z	14.91155	170.0955711	CSID81836	N-Methyl-L-histidine	0.04409952	1.5751343	0.65547484	up
14.96_590.3112n	14.9564	589.3039231	HMDB0004158	Urobilinogen	0.007035755	1.5572509	-0.63900137	down
14.97_283.1551m/z	14.97355	283.1551425	CSID74854303	L-Glutaminyl-L-Arginine	6.47E-04	1.7100081	-0.7740032	down
15.00_445.2493m/z	15.00213333	445.2493131	CSID25937160	3α,7α,12α,16α-tetrahyd	0.004847816	1.6763755	-0.74534535	down
15.02_574.2148n	15.01926667	573.2075643	HMDB0006825	Tetrahydrofolyl-[Glu](2)	0.003804523	1.8973418	-0.92397964	down
15.03_485.3411m/z	15.02593333	485.3410809	HMDB0118021	CL(8:0/10:0/a-25:0/a-25:	0.047622293	1.6458617	0.71884316	up
15.12_251.1447m/z	15.12211667	251.1446756	HMDB0000145	Estrone	0.004216556	2.4895794	1.315902	up

15.14_333.2085m/z	15.13926667	333.208538	HMDB0002122	Prostaglandin F3a	0.036308646	1.5187575	-0.6028915	down
15.16_769.4056m/z	15.15641667	769.4055993	HMDB0265413	PA(20:5/20:5-3OH(5,6,15	1.79E-04	1.6166866	-0.69304	down
15.29_311.2267m/z	15.29355	311.2266968	CSID7827800	9(S)-HPODE	0.013064166	1.6694373	-0.7393619	down
15.36_335.2245m/z	15.36211667	335.2244879	HMDB0001483	Prostaglandin F2b	0.004685445	1.7073737	-0.7717789	down
15.39_474.2086m/z	15.38596667	474.2086253	HMDB0273156	PGP(PGJ2/18:3)	0.026864978	1.522652	0.6065863	up
15.40_555.2002m/z	15.3964	555.2002363	HMDB0006825	Tetrahydrofolyl-[Glu](2)	0.04734563	1.948762	-0.9625579	down
15.54_289.9208m/z	15.54026667	289.9208403	HMDB0043599	TG(15:0/22:5/16:0)	0.005882935	1.761599	-0.8168855	down
15.55_493.3555m/z	15.55068333	493.3555073	HMDB0297242	DG(18:3(10,12,15)-OH(9)	0.002789387	3.0884347	1.6268758	up
15.57_335.2258m/z	15.56783333	335.2257787	HMDB0001442	Prostaglandin E1	0.003637792	1.7899964	-0.83995664	down
15.61_390.3349m/z	15.60883333	390.3349393	CSID30776715	11Z-Octadecenylcarnitin	0.00757697	1.5923336	0.6711426	up
15.68_534.3608m/z	15.67741667	534.3607689	HMDB0011492	LysoPE(0:0/22:2)	0.02603176	1.771321	0.8248257	up
15.69_481.2820m/z	15.68783333	481.2820276	CSID35015985	L-Threonyllysyl-L-prolyl-L	0.007242425	1.6133037	-0.690018	down
15.69_705.3742m/z	15.69456667	705.3742388	HMDB0116518	PGP(a-13:0/i-12:0)	0.039644644	1.5216455	0.6056323	up
15.69_931.6036n	15.68783333	930.5962916	HMDB0289079	PC(22:6-2OH/DiMe(11,5)	0.037818264	1.5493548	-0.63166755	down
15.77_407.2817m/z	15.77353333	407.2817015	HMDB0000419	3b,7a,12a-Trihydroxy-5b	0.024920136	1.6973178	0.7632567	up
15.77_454.2942n	15.77353333	453.2869386	HMDB0296940	DG(20:3-2OH(5,6)/2:0/0:	0.03835908	1.5772458	0.6574075	up
15.79_816.5678n	15.79171667	839.5569962	HMDB0290518	SM(d18:1/6 keto-PGF1al	0.019101772	2.2417703	1.1646384	up
15.84_391.2869m/z	15.84211667	391.2869357	CSID29131	Ursodeoxycholic acid	0.009280845	1.7121753	-0.7758304	down
15.84_438.2993n	15.84211667	437.2920351	HMDB0297051	DG(2:0/20:3(6,8,11)-OH(	0.008242647	1.7699916	-0.8237425	down
15.91_444.2175m/z	15.91068333	444.2174626	HMDB0011471	LysoPE(0:0/14:1)	9.53E-04	1.5716854	-0.6523125	down
16.02_265.1816m/z	16.02496667	265.1815674	HMDB0060055	Tetranor 12-HETE	0.024562092	1.5650793	-0.64623576	down
16.02_418.2231m/z	16.02496667	418.2231169	HMDB0241393	Tetradeca-2,4-dienedioyl	0.00759936	1.5094647	-0.59403706	down
16.02_785.4373m/z	16.02496667	785.4372534	HMDB0264544	PA(19:2/PGJ2)	0.003821015	1.5255303	-0.60931087	down
16.12_615.4570m/z	16.11761667	615.4569704	HMDB0295255	DG(15:0/PGJ2/0:0)	0.03837331	1.5917228	0.67058915	up
16.45_799.4529m/z	16.45353333	799.4529103	HMDB0264999	PA(20:3/20:4-2OH)	5.95E-04	1.6428658	-0.7162146	down
16.47_806.5851m/z	16.47068333	806.5851364	HMDB0290218	CE(LTE4)	0.019195016	2.8465815	-1.5092304	down
16.49_525.3749m/z	16.48781667	525.374914	CSID115267099	N-(3-Hydroxy-15-methyll	0.001854017	1.8214052	-0.86505187	down
16.54_738.5293m/z	16.53925	738.5293041	CSID24766663	PC(16:0/P-16:0)	0.004886203	1.5962318	-0.67467016	down
16.54_792.4900m/z	16.53925	792.4899967	HMDB0261443	PE(20:4-3OH/18:2)	0.007658529	1.9276978	-0.94687885	down
16.56_1047.5897m/z	16.5564	1047.589657	HMDB0274773	PGP(a-25:0/PGE1)	0.014281583	1.7224013	-0.78442127	down
16.56_806.5120m/z	16.5564	806.5119881	HMDB0113476	PE-NMe(20:4/22:6)	3.68E-04	1.7393585	-0.7985553	down
16.56_874.5028m/z	16.5564	874.5028225	HMDB0284321	PE(22:5-O(16,17)/22:5)	0.008360573	2.1651566	-1.1144713	down
16.56_979.6014m/z	16.5564	979.6014104	HMDB0276024	PGP(i-24:0/20:3(6,8,11)-(	0.020224841	1.5144794	-0.59882194	down
16.61_820.5420m/z	16.60781667	820.5420392	HMDB0289657	PC(P-18:1/PGE2)	0.021059707	1.6455169	-0.7185408	down
16.66_378.2430m/z	16.65925	378.2430406	HMDB0000277	Sphingosine 1-phosphate	0.001189006	1.549945	-0.632217	down
16.70_673.5233m/z	16.69503333	673.523294	HMDB0007469	DG(20:3/22:6/0:0)	0.034610577	1.6369916	0.71104693	up
16.71_747.5481m/z	16.71218333	747.5480634	HMDB0296455	DG(21:0/20:4-OH/0:0)	0.033190206	1.5898931	0.66892976	up
16.80_736.5182m/z	16.79638333	736.51819	HMDB0007898	PC(14:0/P-18:1)	0.029406292	1.6123942	-0.6892045	down
16.82_728.4497m/z	16.81503333	728.449699	HMDB0280887	PS(14:0/18:3(10,12,15)-(	0.04082652	1.5773349	0.657489	up
16.85_874.5622m/z	16.84781667	874.5622038	HMDB0283991	PE(22:6-2OH/22:2)	0.006024883	1.9570612	-0.96868885	down
16.97_286.2368m/z	16.9694	286.2368218	HMDB0061656	3-Hydroxytetradecanoic	0.045250595	1.6048292	-0.6824197	down
16.97_320.2380n	16.96781667	319.2307594	HMDB0011134	5-HETE	0.00473435	1.5408114	-0.6236903	down
16.97_768.5570m/z	16.96781667	768.5570377	HMDB0261875	PE(18:1-O/20:0)	0.010740837	2.3707936	1.24537	up
17.05_239.1687m/z	17.04781667	239.1686859	CSID12630	Tetradecanedioic acid	4.41E-04	1.6811986	-0.74949014	down

17.06_758.5026m/z	17.06496667	758.5026008	HMDB0260821	PE(20:3-2OH(5,6)/15:0)	1.11E-04	1.9888633	-0.99194413	down
17.07_413.2657m/z	17.07225	413.2657432	HMDB0241230	Dodec-6-enedioylcarnitir	0.02814506	1.5780616	0.6581535	up
17.17_535.2956m/z	17.16781667	535.2956317	HMDB0242023	N-Docosahexaenoyl Tryp	3.75E-04	1.7262905	0.7876752	up
17.17_894.5496m/z	17.16781667	894.5496168	HMDB0286454	PC(TXB2/18:1)	0.00778772	1.796045	0.8448234	up
17.20_824.5484m/z	17.2021	824.5484237	HMDB0285371	PE(PGF1alpha/P-18:0)	0.020009123	2.0650053	1.0461454	up
17.22_1030.6301m/z	17.21925	1030.630098	HMDB0270979	PG(a-25:0/LTE4)	0.001703991	1.7671009	0.82138443	up
17.29_303.2368m/z	17.28781667	303.2367903	CSID392692	Arachidonic acid	5.59E-04	1.9443179	0.95926416	up
17.29_345.2479m/z	17.28781667	345.2478507	CSID4436580	16,16-Dimethylprostagla	0.003810106	1.546448	0.6289583	up
17.29_466.3186n	17.28781667	487.2932264	HMDB0241986	N-Linoleoyl Tryptophan	2.06E-04	1.7445095	0.8028214	up
17.29_750.5494m/z	17.28781667	750.5494267	HMDB0009445	PE(20:4/P-18:0)	0.016415423	2.0176635	-1.0126855	down
17.29_776.5557m/z	17.28781667	776.555713	HMDB0009266	PE(20:1/20:3)	0.005903326	1.8483397	0.8862299	up
17.39_600.3238m/z	17.39066667	600.3238027	HMDB0288844	PC(2:0/20:3-2OH(5,6))	0.01354588	1.6680671	-0.7381773	down
17.41_495.7495m/z	17.40781667	495.7494525	HMDB0290819	CDP-DG(16:0/18:1-O)	0.020129798	2.1426706	-1.09941	down
17.44_1198.7685m/z	17.44388333	1198.768487	HMDB0242417	Deoxycholyltryptophan	0.043987982	1.5747417	0.65511525	up
17.46_413.3273m/z	17.45925	413.3272633	HMDB0092907	DG(8:0/13:0/0:0)	0.03573414	1.6740295	-0.74332494	down
17.55_711.5276m/z	17.54686667	711.5275909	HMDB0115070	PA(20:0/18:2)	0.04101323	1.6366522	0.7107478	up
17.63_534.3195m/z	17.63066667	534.3195494	HMDB0252830	Gly-Pro-Arg-Pro-Lys	7.10E-05	2.0940244	1.0662782	up
17.73_826.5609m/z	17.73351667	826.560904	HMDB0261913	PE(PGD1/20:0)	0.009569609	2.066423	1.0471356	up
17.75_766.5424m/z	17.75066667	766.5423539	HMDB0286156	PC(18:2+=O(13)/17:0)	0.019493807	1.5761011	0.65636003	up
17.92_675.5378m/z	17.92413333	675.5378422	HMDB0290165	Cer(t18:0/PGJ2)	0.013840365	1.7963321	0.8450541	up
18.00_443.1999m/z	18.0021	443.1999269	CSID4574282	6-dehydrotestosterone 1	0.022189088	1.6449612	-0.7180536	down
18.04_188.5876m/z	18.03853333	188.5876005	HMDB0241053	3-methyloctanedioylcarn	0.015383177	1.5125028	0.5969378	up
18.10_1027.7711m/z	18.10495	1027.771097	HMDB0053548	TG(20:2n6/22:4/22:6)	0.015598347	1.5556755	-0.6375412	down
18.19_597.4167m/z	18.19066667	597.416722	HMDB0294624	DG(20:4-OH(20)/0:0/12:(	0.042454578	1.637902	0.71184903	up
18.24_377.2236m/z	18.24435	377.2235583	HMDB0114829	PA(15:0/22:5)	0.019889608	1.5202392	0.60429835	up
18.26_1091.7214m/z	18.25923333	1091.721416	HMDB0004891	Ganglioside GA2 (d18:1/:	0.04327728	1.515624	-0.5999119	down
18.26_1159.7089m/z	18.25923333	1159.708851	HMDB0011929	Ganglioside GM3 (d18:1/	0.043203637	1.5030584	-0.5879011	down
18.29_697.4493m/z	18.29351667	697.4492862	HMDB0263165	PA(20:3-O(5,6)/15:0)	0.014692624	1.6492959	0.7218503	up
18.29_759.4169m/z	18.29351667	759.416917	HMDB0116572	PGP(i-14:0/i-15:0)	0.04513775	1.5696528	0.65044546	up
18.31_473.1529m/z	18.31293333	473.1529124	HMDB0301002	3-OxoUndecanoyl-CoA	0.026679723	1.6492418	0.72180295	up
18.36_457.2675m/z	18.36435	457.2674806	HMDB0272543	PGP(20:3-2OH(5,6)/18:0)	0.00430206	1.5979414	-0.6762145	down
18.50_549.6684m/z	18.5016	549.6683819	HMDB0011656	2,4,7,10,13,16,19-Docosa	0.01296794	1.7493405	-0.80681115	down
18.55_269.1111m/z	18.55303333	269.1111221	HMDB0266541	PA(2:0/18:1-O)	0.04887398	1.7551763	0.81161594	up
18.69_757.5339n	18.6878	738.5161069	HMDB0261253	PE(18:1-O(12,13)/18:1)	0.017006744	1.5787288	0.65876335	up
18.72_849.5734n	18.72208333	870.5480996	HMDB0282100	PS(18:1-2OH(9,10)/20:0)	0.009487489	1.8545364	0.89105856	up
18.79_1001.5157n	18.79065	1000.508387	HMDB0115969	CDP-DG(18:2/18:2)	0.024781633	1.8191069	0.86323035	up
18.81_804.5744m/z	18.8078	804.5744067	HMDB0112709	PS(22:0/15:0)	0.015871027	1.6054237	0.6829541	up
18.82_1025.5438n	18.82493333	1006.525974	HMDB0293001	CDP-DG(a-17:0/18:1-2OH	0.021550685	1.5483145	0.6306985	up
18.82_1068.4946m/z	18.82493333	1068.494627	HMDB0292983	CDP-DG(a-17:0/20:4-2OH	0.02673066	2.056641	1.04029	up
18.82_1074.5133m/z	18.82493333	1074.513285	HMDB0291418	CDP-DG(PGF2alpha/18:2	0.04091238	1.6821237	0.7502838	up
18.84_959.6031m/z	18.83898333	959.6031071	HMDB0284926	PE(DiMe(13,5)/22:6-2OH	0.003765622	1.6612451	-0.73226494	down
18.84_986.5969m/z	18.83898333	986.5969217	HMDB0276996	PI(PGJ2/20:0)	0.003069517	1.5890013	-0.66812027	down
19.26_463.7392m/z	19.26308333	463.7391573	HMDB0282764	PS(PGD1/20:4)	0.032147795	1.5534552	-0.63548064	down
19.28_307.1626m/z	19.27753333	307.1625846	CSID5898	Androstenedione	0.009189721	2.1289303	-1.0901288	down

19.31_1007.5005n	19.31451667	972.486614	HMDB0293508	CDP-DG(i-14:0/20:3-2OH	0.007183861	1.9877077	-0.9911056	down
19.35_1000.5101m/z	19.3488	1000.510065	HMDB0291229	CDP-DG(18:1/18:2+=O(9)	0.026381325	1.7028009	-0.7679097	down
19.45_218.5758m/z	19.45176667	218.5758404	HMDB0012575	13E-Tetranor-16-carboxy	0.023378428	2.1023138	1.071978	up
19.45_486.1507m/z	19.45176667	486.1507454	HMDB0012607	18-Carboxy-dinor-LTE4	0.04960649	1.9841218	0.9885006	up
19.47_336.2187m/z	19.4689	336.2187331	HMDB0262853	PA(20:3-O(5,6)/13:0)	0.031121302	1.8469414	0.88513803	up
19.47_393.2178m/z	19.4689	393.2178016	CSID4446239	19(R)-hydroxy Prostaglar	0.017506853	1.8912548	0.9193437	up
19.50_328.3184m/z	19.50318333	328.318357	HMDB0062673	2-(4-methyl-1-piperaziny	0.007052444	3.0262184	1.5975161	up
19.72_813.6730m/z	19.72043333	813.6730068	HMDB0042329	TG(14:0/16:1/22:6)	0.030120747	1.5237108	0.6075891	up
19.72_949.6051m/z	19.72043333	949.6051298	HMDB0284580	PE(24:1/LTE4)	0.001119191	1.6130213	-0.68976545	down
19.81_465.3121m/z	19.80895	465.3121398	HMDB0241986	N-Linoleoyl Tryptophan	0.03445318	1.6291856	0.7041509	up
19.83_762.5156m/z	19.8261	762.51558	HMDB0261501	PE(20:3(6,8,11)-OH(5)/18	0.022530861	1.6376882	0.7116607	up
19.99_410.7577m/z	19.99483333	410.7577083	HMDB0285824	PC(15:0/PGJ2)	0.00598081	1.633424	0.70789933	up
2.46_400.0822m/z	2.459516667	400.0822031	HMDB0001066	S-Lactoylglutathione	0.028707381	1.6492099	-0.721775	down
2.63_428.1186m/z	2.63095	428.1186225	CSID35014630	gamma-Glutamyl-gamma	0.004177824	1.7101542	-0.7741264	down
2.65_247.0439m/z	2.648083333	247.0439048	HMDB0240292	2,3,4,5,6,7-Hexahydroxyl	4.88E-05	2.9993691	-1.5846591	down
2.68_130.0857m/z	2.682366667	130.0857363	CSID834	Leucine	0.004606862	1.628603	-0.7036349	down
2.68_293.1487n	2.682366667	292.1414609	HMDB0241652	3,4,5-Trihydroxypentano	0.007287748	1.7056208	-0.7702969	down
2.70_244.1219m/z	2.699516667	244.1219497	HMDB0241651	3-Oxobutanoylcarnitine	0.004386674	1.7710193	-0.82457995	down
2.70_486.1604m/z	2.699516667	486.1603969	HMDB0003324	Biotripyrrin-b	0.006709321	1.7675397	-0.82174265	down
2.80_380.0988m/z	2.802366667	380.0988481	HMDB0010336	Epinephrine glucuronide	1.09E-04	2.6057134	-1.3816783	down
3.22_315.1917m/z	3.21975	315.1917087	HMDB0241670	3-Hydroxyhex-4-enoylcar	0.008641013	1.946545	-0.9609157	down
3.43_236.0951m/z	3.430933333	236.0950907	HMDB0062175	N-Lactoylphenylalanine	0.007019697	1.6513504	-0.7236462	down
3.45_239.0135m/z	3.448066667	239.0134942	CSID575	cystine	1.42E-04	2.7568254	1.4630079	up
3.52_323.0415m/z	3.516633333	323.0414887	HMDB0060017	Pyrogallol-2-O-glucuronic	0.040874436	1.7289021	0.7898562	up
4.09_323.0414m/z	4.093766667	323.0413751	HMDB0060017	Pyrogallol-2-O-glucuronic	0.003501365	2.2296894	1.1568427	up
4.16_456.2083m/z	4.156866667	456.2083045	HMDB0003752	LysoPC(10:0/0:0)	0.001171011	1.7388389	-0.79812425	down
4.19_114.0667m/z	4.19115	114.0667057	HMDB0000064	Creatine	0.046039097	1.5991646	-0.67731845	down
4.76_241.0315m/z	4.762316667	241.031549	HMDB0059775	3-Hydroxy-3-carboxymet	0.010624834	2.0082293	1.005924	up
4.83_240.0904m/z	4.830883333	240.0904384	HMDB0013134	Fumarycarnitine	0.033202693	1.837562	-0.8777929	down
5.23_375.1874m/z	5.231133333	375.18742	HMDB0241866	8-Amino-7-oxononanoyl	0.025109237	1.5224904	-0.6064331	down
5.77_233.0966m/z	5.774	233.0966139	HMDB0000670	Homo-L-arginine	5.23E-06	2.0149727	-1.0107603	down
5.80_437.0908m/z	5.8023	437.0907654	HMDB0001520	Flavin mononucleotide	5.67E-04	1.9248943	-0.9447792	down
6.09_290.0678m/z	6.088	290.0677578	HMDB0010319	Indoxyl glucuronide	0.006190945	1.5719658	-0.65256983	down
6.68_348.1897m/z	6.67685	348.1897167	HMDB0275649	PGP(LTE4/i-20:0)	0.021746377	2.525283	-1.3364451	down
9.40_358.0541m/z	9.3965	358.0541199	HMDB0013189	3-Indole carboxylic acid g	0.004395961	1.881724	-0.912055	down
