# Supplementary figures and images for: Metabolomic Effects of Liraglutide Therapy on the Plasma Metabolomic Profile of Patients with Obesity
Source: Metabolites. 2024 Sep 17;14(9):500. doi: 10.3390/metabo14090500 (PMC11433991; doi:10.3390/metabo14090500)

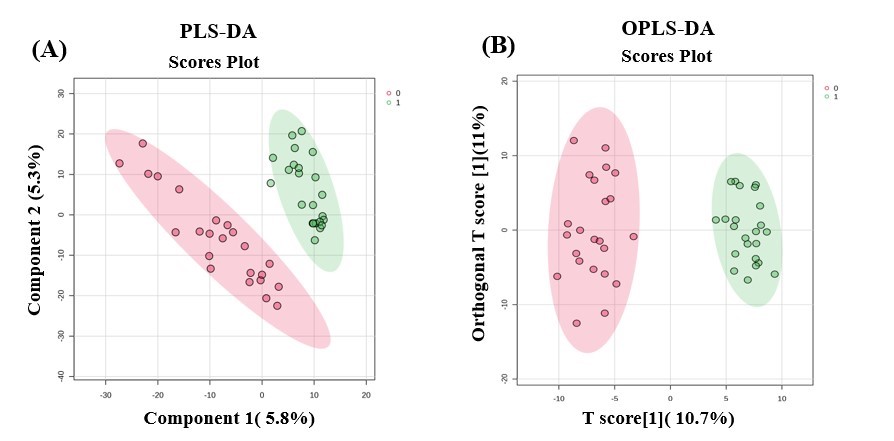

Supplement: Supplementary file 1 [file metabolites-14-00500-s001.zip › Supplimentary Figure 1.jpg]

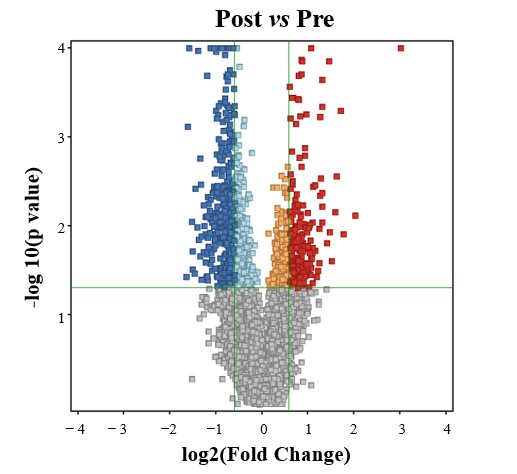

Supplement: Supplementary file 1 [file metabolites-14-00500-s001.zip › Supplimentary Figure 2.jpg]

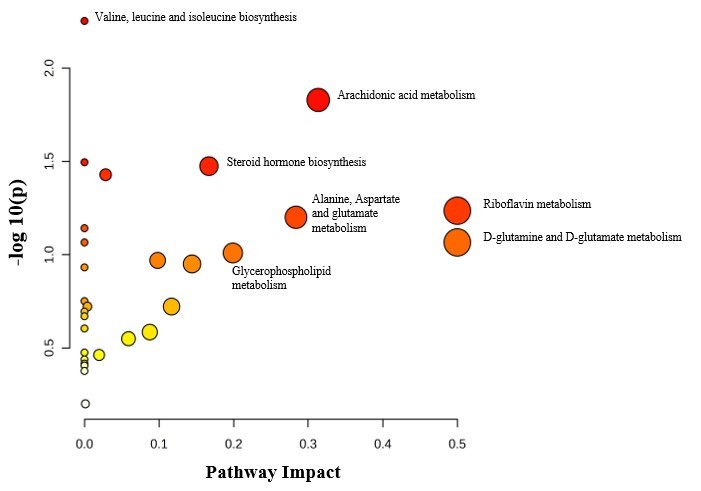

Supplement: Supplementary file 1 [file metabolites-14-00500-s001.zip › Supplimentary Figure 3.jpg]
